# The Development of Efficient Contaminated Polymer Materials Shredding in Recycling Processes

**DOI:** 10.3390/polym13050713

**Published:** 2021-02-26

**Authors:** Józef Flizikowski, Weronika Kruszelnicka, Marek Macko

**Affiliations:** 1 Department of Machines and Technical Systems, Faculty of Mechanical Engineering, University of Science and Technology in Bydgoszcz, Kaliskiego 7 Street, 85-789 Bydgoszcz, Poland; 2 Department of Mechatronic Systems, Faculty of Mechatronics, Kazimierz Wielki University, Chodkiewicza 30 Street, 85-064 Bydgoszcz, Poland

**Keywords:** shredding, mechanical recycling, contaminated polymeric materials

## Abstract

Recently, a dynamic increase in the number of polymer elements ending their life cycle has been observed. There are three main ways of dealing with polymer waste: reuse in an unchanged form, recycling (both material and energy), and disposal (mainly in the form of landfilling or incineration). The legislation of European countries promotes in particular two forms of waste management: reuse and recycling. Recycling processes are used to recover materials and energy especially from contaminated waste, which are structurally changed by other materials, friction, temperature, machine, process, etc. The recycling of polymers, especially of multi-plastic structural elements, requires the use of special technological installations and a series of preparatory operations, including crushing and separating. Due to the universality and necessity of materials processing in recycling engineering, in particular size reduction, the aim of this study is to organize and systematize knowledge about shredding in the recycling process of end-of-life polymeric materials. This could help properly design these processes in the context of sustainable development and circular economy. Firstly, an overview of the possibilities of end-of-life plastics management was made, and the meaning of shredding in the end-of-life pathways was described. Then, the development of comminution in recycling processes was presented, with special emphasis given to quasi-cutting as the dominant mode of comminution of polymeric materials. The phenomenon of quasi-cutting, as well as factors related to the material, the operation of the shredding machine, and the technological process affecting it were described. Research conducted on quasi-cutting as a phenomenon when cutting single material samples and quasi-cutting as a machine process was characterized. Then, issues regarding recycling potentials in the context of shredding were systematized. Considerations included the areas of material, technical, energy, human, and control potentials. Presented bases and models can be used to support the innovation of creative activities, i.e., environmentally friendly actions, that produce specific positive environmental results in the mechanical processing of recycled and reused materials. The literature survey indicates the need to explore the environmental aspect of the shredding process in recycling and connect the shredding process variables with environmental consequences. This will help to design and control the processes to get the lowest possible environmental burdens.

## 1. Introduction

In the last decade, there has been a growing interest in the issues of optimal choice of materials processing parameters and technologies providing high efficiency and low environmental impact [1,2,3,4,5]. The carried out research and analyses as well as technological development have resulted in a dynamic growth of plastics consumption in almost every field: packaging, the aviation and automotive industries, and power engineering [6,7]. The problem is the post-use management of plastics. The amount of plastic waste has been increasing year by year, while the methods of its segregation, processing, and recovery are limited. The limitations in processing are mainly caused by the variability of properties of materials forming the waste stream and technological possibilities of processing equipment [6,8,9,10,11].

The actions implemented to protect the climate and increase energy efficiency have led to a rapid development and increase in the number of functioning photovoltaic and wind installations in which polymeric materials are the main building material [8,12,13,14]. Due to a relatively short period of development of these installations, no effective method of management and processing of polymeric materials into end-of-life (EoL) stages has been developed so far [8,15,16,17,18]. The post-use materials and components of wind power plants and PV modules are most often stored or burned with energy recovery in specially designed installations for the processing of polymeric waste [8,15,16,17,18]. While various forms of mechanical, chemical, and thermal recycling are used in recycling, they are still at the initial stage of development, and it is necessary to develop machines, devices, and technologies based on innovative solutions that are highly effective and consistent with the idea of eco-design [8,19,20].

Environmental assessment at the EoL stage should also be an inseparable element of the analyses, especially considering that the main raw materials for the production of polymeric materials are oil and gas, the extraction of which exhausts the available resources and causes negative environmental impacts [8,10]. The recycling of plastics makes it possible to significantly reduce the environmental burden during the life cycle and to recover some of the natural resources and energy [21,22]. Previous studies [14,18,22,23,24,25,26,27,28] have shown that for multi-plastic components from which it is difficult to separate individual materials (e.g., wind turbine blades, automobile tires), the use of recycling as a form of post-consumer management significantly contributes to the reduction of environmental impacts during the life cycle of these components.

**Figure 1:**
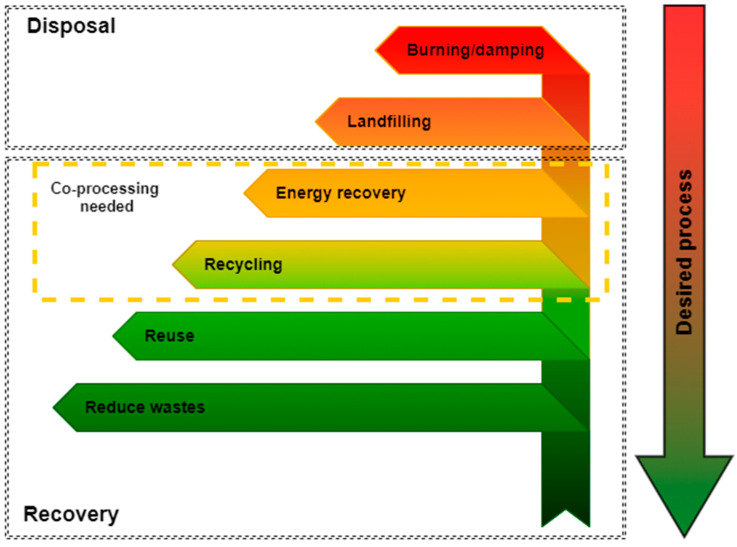
Polymers waste end-of-life (EoL) options based on the EU Waste Framework Directive.

The EU Waste Framework Directive explicitly indicates the guidelines for the treatment of waste, including polymeric waste, emphasizing waste treatment during which it is possible to recover as much raw material and energy as possible (Figure 1) [29,30].

The reprocessing of materials in recycling requires their initial preparation [6]. The most frequently used operations are segregation, cleaning, or washing and shredding, which is particularly important in the case of processing large-size polymeric materials [6,9,18,31]. Shredding processes are in principle an integral part of recycling; therefore, their efficiency should be as high as possible, and their energy consumption should be as low as possible, in order to have an adequate balance between the energy and environmental inputs for the processing of recycled materials, and the benefits of raw material and energy recovery [32,33,34]. In this case, it is necessary to recognize the relations between the shredding parameters, design features of shredders, and the properties of polymeric materials. The mentioned relations will influence the process efficiency and the obtained product quality as well as the energy consumption during the shredding process. Issues regarding relationships in material–machine–process areas in the case of polymeric materials shredding have not yet been sufficiently addressed [35,36,37,38,39,40,41,42,43,44]. In this paper, an attempt has been made to describe the indicated issues in the aspect of polymeric materials recycling.

There are many publications devoted to specific, single aspects of polymeric materials shredding [6,9,45,46,47,48,49,50,51,52], but there is a lack of study summing up and connecting different aspects of shredding in the recycling process, dealing with relation between shredded material, machine features, process indicators, and the environment. These relations will be fundamental for shredding process development.

The aim of this study is to organize and systematize knowledge about shredding in the recycling process of end-of-life polymeric materials, which could help properly design these processes in the context of sustainable development and circular economy. It examines the latest literature data on different shredding technologies in the recycling of polymeric material as well as recommendations for the eco-design of energy–material systems. In this study, we provide fundamentals for the mathematical description of shredding in the recycling engineering including energy–material potential, energy relations, and efficiency indicators. The issues of efficiency, control, optimization, and modeling of the end-of-life polymeric materials shredding process were presented. These were considered for the special construction of a multi-hole shredding unit, taking into account the following variables: machine design features, movement indicators, changes in the volume of material, and cutting surface during shredding.

The remainder of this paper is structured as follows (besides this introduction): Section 2 covers the issues of the possibility of managing end-of-life polymer materials and the importance of shredding in recycling; Section 3 presents phenomenological attitudes of shredding along with disturbances and factors influencing the course of the process and literature review in the field of machine research of the polymer materials shredding; Section 4 organizes the aspects of the polymer materials recycling potential in relation to the shredding processes, taking into account human, technological, energy–material and control resources; the last section contains a summary and the conclusion.

## 2. Recycling Engineering and Shredding

Recycling engineering describes the areas of activity connected with the designing of the EoL and processing of the EoL materials. It includes issues of design for recycling as well as process and materials engineering on the complete life cycle stages of objects.

In recycling engineering, there are four basics principles of the development and potential of recycling processes control. The first basic principle is high quality and development of polymeric construction materials that are easy to recycle. The second is efficiency (energy and material) of the processing and use of these materials. The third one is the harmlessness of products and processes throughout their life cycle. Finally, the fourth principle is constant development of knowledge about polymer engineering and recycling pathways [9,53,54].

### 2.1. End-Of-Life Options for Contaminated Polymeric Materials

With the development of technology, many methods of recovery, processing, and management of end-of-life contaminated, structurally changed polymeric materials have appeared over the years [55]. The universality of a given management technology is influenced by, among others, the current legislation, the cost of its implementation, and the environmental impact. Currently, the most important legal acts covering the issues of handling polymer waste include the following:

- Waste Framework Directive (Directive 2008/98/EC on waste) [29],
- Council Directive 1999/31/EC of 26 April 1999 on the landfill of waste [56],
- Directive (EU) 2018/852 Of The European Parliament and of The Council of 30 May 2018 amending Directive 94/62/EC on packaging and packaging waste (Text with EEA relevance) [57],
- Directive 2000/53/EC of the European Parliament and of the Council of 18 September 2000 on end-of-life vehicles [58],
- Directive 2012/19/EU of the European Parliament and of the Council of 4 July 2012 on waste electrical and electronic equipment (WEEE) [59].

These documents define, inter alia, the method of collection, segregation, processing, or EoL management, along with an indication of preferred methods ensuring the possibly highest share of material and energy recovery and the possibly lowest generation of environmental burdens, e.g., in the form of dust, harmful substances, noise, etc.

**Figure 2:**
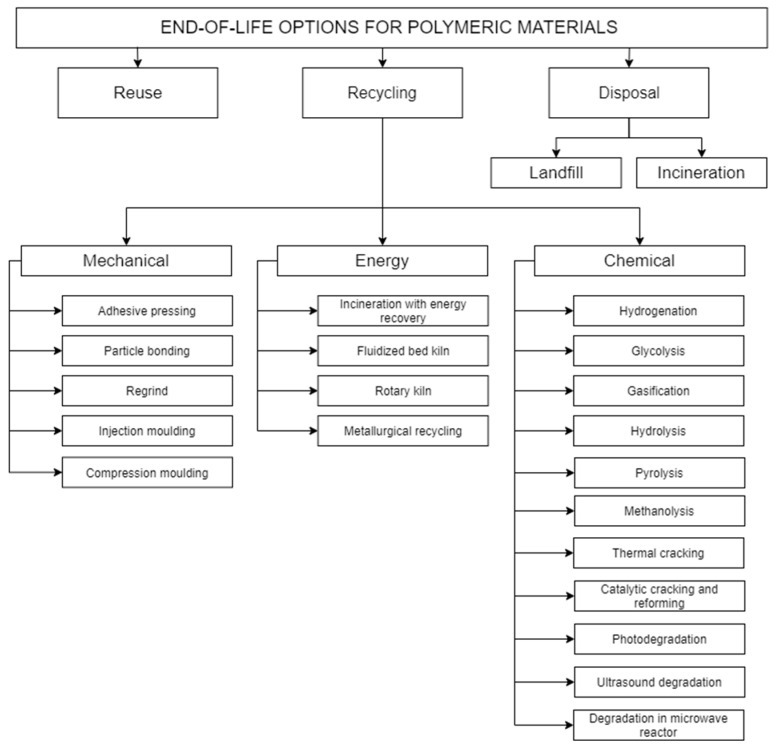
End-of-life options for contaminated, structurally changed plastic waste. Own work based on data from [62,63,64,65,66,67,68,69].

According to the EU Waste Framework Directive (Figure 1), the amount of generated waste should be reduced first (e.g., through a conscious design of products consisting of minimizing the consumption of raw materials, facilitating reuse and recycling) [29,54,60,61]. With regard to plastic waste, four main methods of EoL management (Figure 2) are used: reuse, recycling, landfilling, and incineration (without energy recovery).

**Figure 3:**
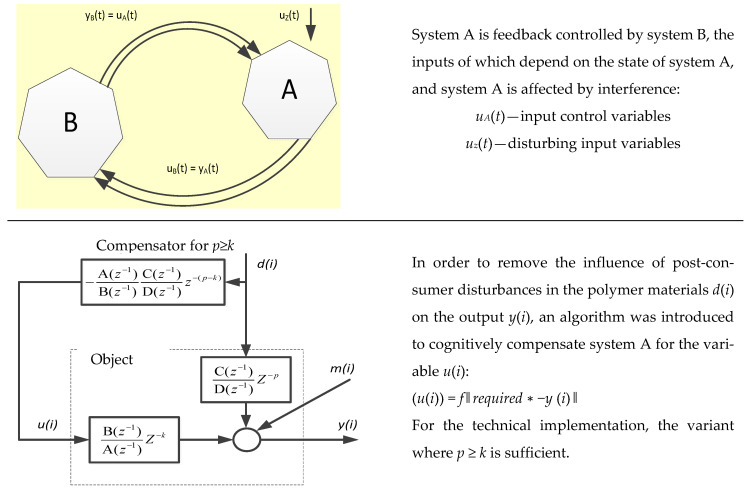
Control, regulation, and compensation of interference of polymer materials resulting from operation. Own work based on data from [70,71,72].

Most often, after the use phase, polymer materials have disturbances (contamination, changes) (system A) of the internal and external structure (shape), filling with other materials, substances, pollutants, etc. [62]. Waste processing technologies can be implemented under the condition of appropriate control, appropriate regulation, and disturbance compensation. Waste treatment system control requires defining the input function in a way that ensures the achievement of the set goal (Figure 3). Processing system A is affected by disturbances and is controlled with feedback by system B, the inputs of which depend on the disturbance state of the polymer material by waste.

Controlling a waste processing system requires determining an output function *y_A_*(*t*) (control) that depends on process, material, and machine factors (input variables *u_A_*(*t*)) (Figure 3). The output function can be a set of specified parameters of the processing product, assumed performance, or energy consumption level. Maintaining the parameters at the assumed level is realized on the basis of the feedback principle of the connected two systems: processing system A and control system B (Figure 3). If the processing system A is affected by interference *u_z_*(*t*) (e.g., in the form of impurities of the processed materials), which results in deterioration of the set of output parameters *y_A_*(*t*), then system A is controlled with feedback by system B, in which the values of the output function are analyzed. For system B, the values of the output function of system A *y_A_*(*t*) constitute the input signal *u_B_*(*t*) (input values), based on which the control system B will generate the output signal *y_B_*(*t*) which is also the input signal *u_A_*(*t*) for system A. In this way, the interference is compensated, and it is possible to maintain the output values of system A at the assumed level.

Negative feedback is used for the following purposes:

- Reduce the impact of disturbances in polymer materials from operation, affecting the initial size of the object.
- Reduce the impact of changes in object parameters on its output size.
- Modify the static and dynamic properties of the object

#### 2.1.1. Reuse

The concept of material/product reuse assumes that after the completion of the first life cycle, the product is not discarded (e.g., in the case of bottles after they have been emptied) but begins another life cycle with the same function (e.g., beverage bottles collected by manufacturers are filled again) or a completely different function without any processing operations, e.g., using yogurt containers as flower pots. [29,61]. Most often, in the case of a contaminated (disturbed) polymer waste (system A, Figure 3), certain preparatory operations (e.g., system B, Figure 3) are necessary, such as sorting, washing, drying, or repair. Reuse operations can take place in a closed loop within a single enterprise or company [61]. A system B example would be the refilling of beverage bottles.

#### 2.1.2. Recycling

Recycling is the next way to manage contaminated EoL polymer materials. There are mechanical, energy, and chemical recycling methods (Figure 2), with priority being operations resulting in the highest recovery of materials and energy and the lowest energy demand in processing.

**Figure 4:**
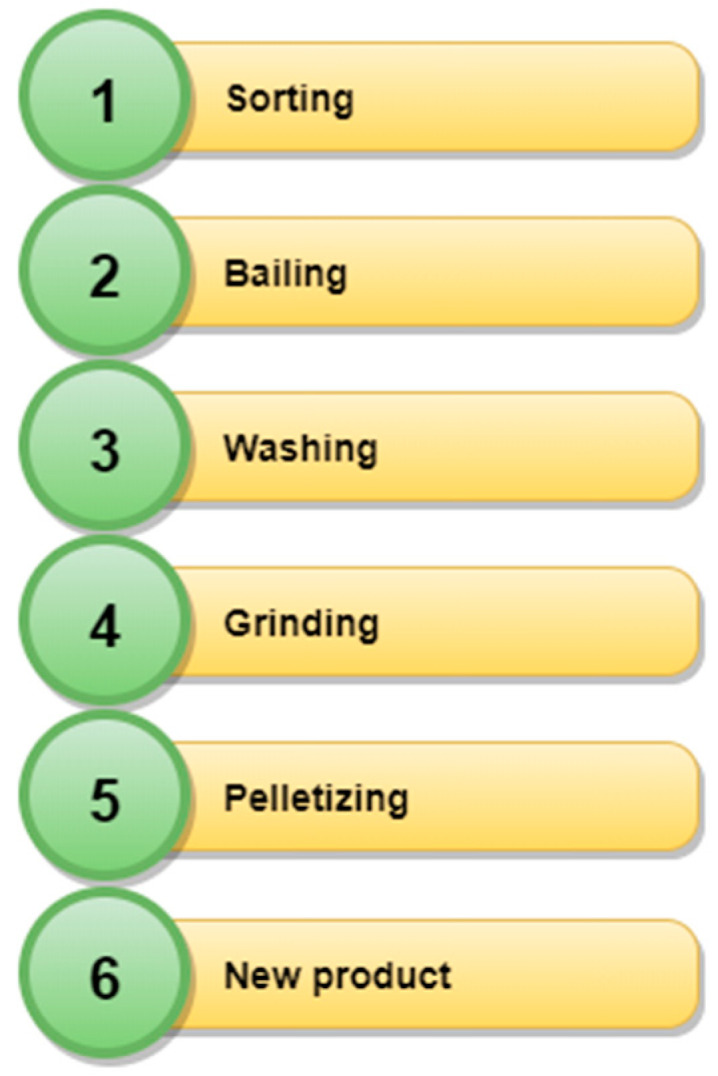
The typical route of contaminated plastics waste processing in mechanical recycling according to [64,65].

Mechanical recycling includes operations of the mechanical processing of polymer waste without interfering with their chemical structure [9,73]. As a result of mechanical recycling, pellets are obtained, which are then used as input material in the creation of new products [9]. Mechanical recycling takes into account the elimination of disturbances, e.g., (Figure 4): sorting, bailing, washing, shredding, pelleting, and the production of a new product [64,73]. The ASTM D7209 standard [74] introduced the notions of primary and secondary recycling, which basically cover activities in mechanical recycling. Primary recycling most often refers to closed-loop recycling in the pre-use phase, when waste directly from production goes back to the extrusion process, e.g., in the case of obtaining incorrectly shaped bottles that do not meet the requirements [64,65]. In turn, secondary recycling concerns post-consumer waste, which is processed in accordance with the diagram in Figure 4, as a result of which new materials are created, often with worse properties [64,65].

Chemical recycling is defined as “a process which converts contaminated polymeric waste by changing its chemical structure to produce substances that are used as raw materials for the manufacturing of new products, which excludes [the] production of fuels or means of energy generation” [75]. There are many technologies for chemical recycling (see Figure 2), and some of them need a high temperature to break the polymer bonds, for example pyrolysis, gasification, and thermal cracking [65,66]. In this case, the waste also requires appropriate preparation. Chemical recycling is also called tertiary recycling [64,65].

Energy recycling or energy recovery (and also quaternary recycling according to ASTM D7209 [74]) covers operations aimed at recovering the energy contained in disturbed polymer materials [64,65]. Energy recovery is most often carried out during waste incineration in waste incineration plants, fluidized bed furnaces, and the metallurgical industry (Figure 2). Then, the amount of polymer waste, which cannot be reprocessed in mechanical recycling and would be deposited in landfills, is reduced. The recovered energy is mainly used to produce heat and electricity [64,65,66,76]. Energy recovery is a controversial method of recycling due to the emissions resulting from the combustion of contaminated polymers, although in recent years, a number of solutions have been introduced to prevent the release of harmful substances during combustion or reduce their amount [66,67,68,69].

#### 2.1.3. Disposal

Disposal is the last option of EoL management of contaminated polymer materials according to the EU Waste Framework Directive [29], and at the same time, it is the least appropriate method of ending the life cycle of products from the point of view of the circular economy. Disposal is defined as [62] “any operation which is not recovery even where the operation has as a secondary consequence of the reclamation of substances or energy”. The two most common disposal methods are landfilling and incineration without energy recovery. In both cases, the material and energy potential is lost because they will not be used (they will not replace the natural, original raw materials) [62,63].

**Figure 5:**
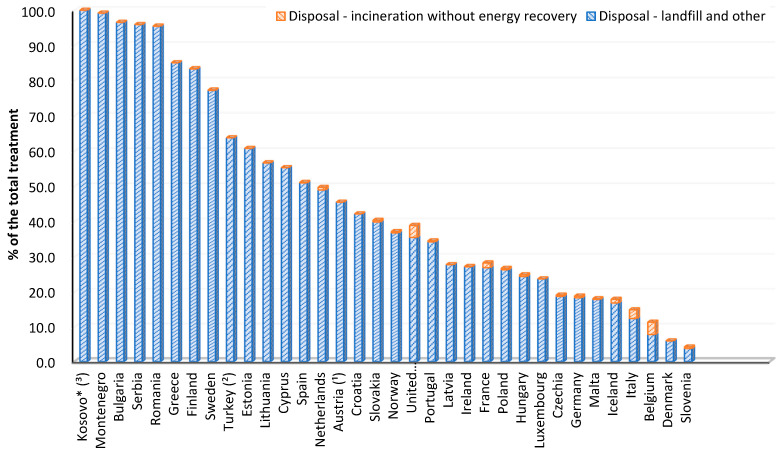
The share of different types of disposal in total treatment options of waste in EU countries in 2018. Own work based on Eurostat data [77].

Landfilling is considered to be the simplest and cheapest method of managing EoL disturbed polymer materials, although it requires large land areas for this purpose. Landfilling may cause soil and groundwater pollution with toxic substances derived from the decomposition of polymer waste. Currently, it is popular to build biogas plants that extract methane from the decomposition of organic polymer waste [63,68,76]. In many EU countries, according to Eurostat data, landfilling is the dominant form of waste management (Figure 5) [77].

The number of contaminated polymeric materials, which was increasing from year to year, and the lack of areas for their storage resulted in the spread of waste incineration. Incineration is primarily aimed at reducing the amount of waste deposited in landfills [67,68,69]. Incineration was a very popular method of waste management, right after landfilling, until 1980, when it was found that incineration plants emit significant amounts of emissions in the form of dioxins and volatile heavy metal compounds, and then air pollution control (APC) technologies were introduced [67]. With the development of technology and the introduction of climate protection directives and waste management strategies, incineration is carried out with energy recovery (energy recycling), and in some countries, the incineration of waste (without energy recovery) has been completely prohibited [68]. In 2018, Belgium (3.6%) had the largest share of incineration without energy recovery in all forms of waste management, which was followed by Great Britain (3.4%) (Figure 5) [77].

### 2.2. End-Of-Life Contaminated Polymer Material Potential and Scale

**Figure 6:**
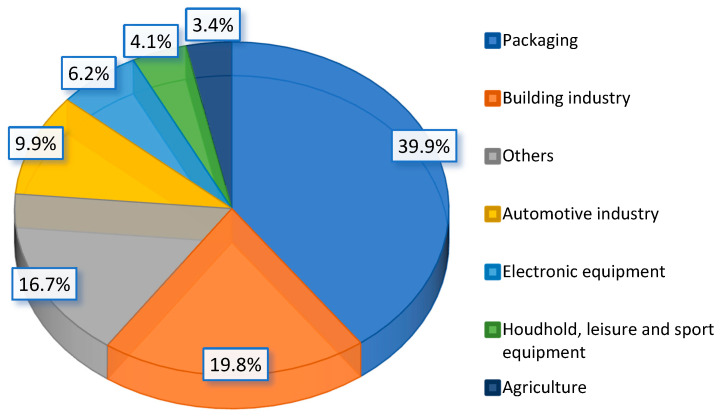
The contribution of different industries in plastics demand in Europe in 2018. Own work based on data published in [78].

Polymer materials are a group of materials with various properties, which allowed them to be widely used in many industries, including construction, food, electronics, automotive, chemical, and gardening industries, of which the largest share is packaging (39.9% in 2018) and building (19.8% in 2018) (Figure 6) [78]. According to the PlasticsEurope report, the total demand for plastics in Europe was 51.2 million tonnes [78].

**Figure 7:**
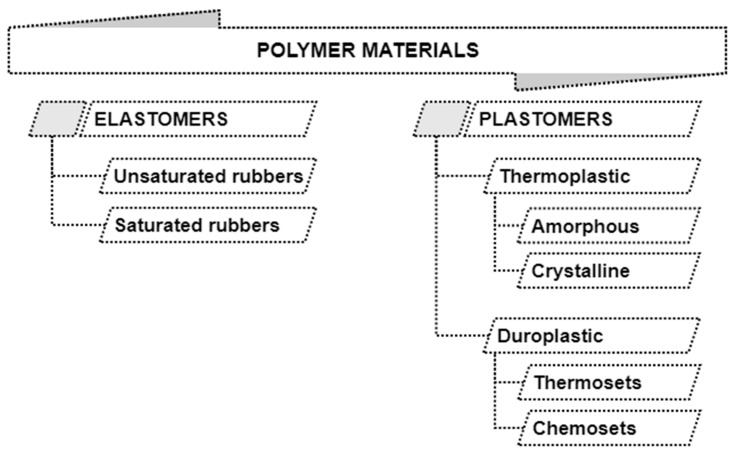
Classification of polymeric materials according to [79,80].

Polymer materials currently available on the market can be classified into elastomers, which are characterized by the reversibility of deformations resulting from the application of loads, and plastomers, which, when deformed, keep their shape, and their deformability is related to the temperature [79,80]. Plastomers are divided into thermoplastics, which soften under the influence of temperature and can be shaped many times, which is an obvious advantage in recycling processes, and duroplasts, which change their chemical structure under the influence of temperature. Special systems of bonds are formed, hardening the structure of the material, which means that they cannot be remelted or reshaped and make reprocessing difficult [79,80]. The full classification of polymers is shown in Figure 7.

The commonly used thermoplastics include the following: Polyethylene (PE), Polyamides (PA), Polypropylene (PP), Polycarbonate (PC), Acrylonitrile butadiene styrene (ABS), Ethylene-vinyl alcohol (EVOH), Styrene acrylonitrile (SAN), Polyether ether ketone (PEEK), Polyoxymethylene (POM), Expanded polystyrene (foamed) EPS, Polysulfone (PSU), Polystyrene (PS), Thermoplastic elastomers (TPE), Polyethylene terephthalate (PET), Polymethyl methacrylate (PMMA), Polyvinyl chloride (PVC), Fluoropolymers [78]. The commonly known thermosets are the following: Phenol–formaldehyde resins, Polysiloxanes (Silicones), Vinyl–ester resins, Polyesters, Urea–formaldehyde resins, Polyurethanes (PUR), Polyimides, Melamine resins, epoxy resins, acrylic resins [78]. The largest percentage of the mentioned plastics used in 2018 was Polypropylene and Polyethylene [78].

**Figure 8:**
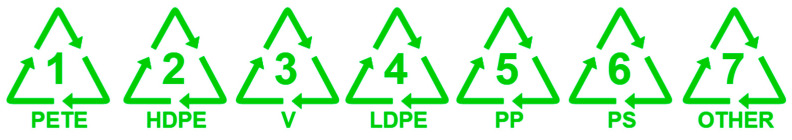
Designations for different groups of plastics in recycling according to [81].

Due to their properties, polymers were divided into seven groups according to types of recyclable waste, which were specially marked for easier identification during sorting and waste collection (Figure 8) [81].

According to PlasticsEurope data, 29.1 million tonnes of contaminated polymer materials were collected, and a further upward trend is observed [78]. The dominant way of waste management was energy recovery (42.6%), followed by recycling (32.5%) and landfilling (24.9%) [82]. Both energy recovery and recycling show an upward trend every year, while a decrease in the number of waste subjected to landfilling is observed [78]. Nevertheless, it is evident how a huge challenge is the management of contamination during the use of polymers materials, which is expressed, among others, by the still significant share of waste storage as well as a large share of energy recovery in the total balance of polymer materials EoL management methods.

To measure the level of recyclability of different materials, the recyclability indicators were proposed by some researchers. Recyclability describes the ability of materials to be recycled with the use of available technologies. In an ideal situation, 100% of a material can be reused, recycled, or regenerated without generating losses in material and energy as well as producing wastes. In fact, bearing in mind actual technological possibilities, the ideal state is not reached. As was presented in a Plastics Europe report, in 2018, 35.5% of polymer waste was recycled, and 42.6% was subjected to energy recovery processes [82]. In total, about 75.1% of all plastics waste was subjected to recycling and energy recovery processes.

One of the indicators proposed by the Organisation for Economic Co-operation and Development (OECD) in the OECD Sustainable Manufacturing Toolkit to measure the level of recyclability is recyclability of products (RoP), which allows estimating the average recyclability of all products manufactured in a company in the reference year [83]. The RoP can be calculated according to the equation:

(1) $$RoP=\frac{\sum W\cdot PRC\cdot UP}{\sum W\cdot UP}\cdot 100\%$$

where *W*—Weight of a product unit, *PRC*—Proportion of recyclable content, *UP*—Units produced. This indicator can take values from 1% to 100%. It does not give information about number of products actually recycled [83].

In report [84], a different understanding of recyclability was proposed. The measure of recyclability was connected with the mass of the products and described by the equation:

(2) $$R_{M}=\frac{X-Y}{X}\cdot 100\%$$

where *R_M_*—recyclability by mass (%), *X*—mass of the recycled product (tonne), *Y*—material losses during recycling process (tonne). The advantage of this indicator is its ease of interpretation and implementation. It gives real information about the value of the product recycled; however, it does not take into account the differences between reused and recycled materials [84].

Other methods describe recyclability by monetary (cost) value. The WRAP report [84] presents two approaches for estimating the recyclability based on value. The first approach defines the difference in cost for processing products from recycling and from primary sources, referring to the cost of the product from primary sources [84]:

(3) $$R_{V}=\frac{Z-Y}{X}\cdot 100\%$$

where *Z*—cost of product B made from primary raw materials ($Z/tonne), *Y*—cost of product B reprocessed from product A ($Y/tonne), *X*—cost of production of product A ($X/tonne).

The second approach describes recyclability as a ratio of product A into product B reprocessing cost ($Y/tonne) and product A production cost ($X/tonne), according to the following equation [84]:

(4) $$R_{V2}=\frac{Y}{X}$$

The biggest drawbacks of assessing recyclability by value are difficulties in the estimation of real/detailed cost of products/material reprocessing, especially if only some percentage of material is reprocessed. The mentioned two approaches do not differentiate the quantities of different savings of material reprocessing [84].

Currently, polymeric materials produced on the basis of recycled materials are determined by the recycled content index, which describes the content of recycled materials in the total weight of the product [85]:

(5) $$RC=\frac{W_{R}+W_{RU}}{W_{IM}}\cdot 100\%$$

where *W_R_*—total weight of recycled material, *W_RU_*—total weight of reused material, *W_IM_*—total weight of material input.

When considering the possibility of recycling the contaminated polymer materials, it should not be forgotten that the recycling processes change the properties of the processed materials. As a rule, deterioration of the functional and mechanical properties of polymers is observed. Some materials are more susceptible to reprocessing (they can be reused and processed several times), and unfortunately, some of them can only be processed once. Bearing in mind the assumptions of the circular economy, it is necessary to determine how many times a given material can be recycled without a significant loss of properties. The following indicator was proposed to assess the recirculation of plastics [86]:

(6) $$R_{1}=R\frac{1-\phi }{1-k\phi }$$

where *R*_1_—initial material property, *ϕ*—the degree of material reprocessing, which determines the mass fraction, which is constantly returned for processing (*ϕ* < 1), *k*—coefficient of deterioration of the material properties, i.e., the number by which one should multiply e.g., the strength *R*_1_ of the material introduced into processing in order to calculate its strength *R*_2_, which it will show after leaving the processing (*k* < 1). This index describes the dependence of the selected property of input material *R*_1_ on this property of material after processing *R*_2_ after a known number of cycles *n*.

### 2.3. Importance of the Shredding Process in Recycling Engineering

To begin with, we consider the scale of contaminated polymer materials shredding. So far, no quantitative analysis of the materials that are subjected to shredding has been performed. This is a complex issue, because the contaminated part of plastics may require prior separation from multi-plastic elements, which is also very often done in comminution and separation processes. The study [48] shows the estimated percentage share of wastes (not only polymer wastes), which are subjected to shredding. Assuming a similar scale, it is possible to estimate the amount of disturbed polymer wastes subjected to shredding depending on the EoL management method. Table 1 shows the estimated scale of polymers shredding in 2018 in the EU countries based on the share of shredded plastics assumed according to [48].

Referring to the estimated data presented in the Table 1, about 25% of plastic waste is shredded annually, which gives over 7 million tons. Globally, this number will increase due to a greater amount of disturbed polymeric materials generated. Only the scale of the process shows the great importance of shredding. It should be emphasized that, for some elements, shredding is a necessary stage in EoL management processes, e.g., in mechanical recycling of car tires [25,87,88,89], recycling of photovoltaic modules [17,18], recycling of wind power blades [16,90,91], or car recycling [92,93,94], where shredding is used to reduce the size and separate the individual materials that make up the elements, so the estimated amount of shredded waste can be much higher. According to the data published in [81], up to 100% of polymer materials are shredded for recycling, which significantly increases the share (up to 41.7%) of end-of-life materials that need to be shredded.

In order to determine the need and importance of comminution processes in recycling, or in the management of contaminated polymeric materials ending their life cycle, it is necessary to ask and answer the basic question: what is the purpose of the treatment of reducing the dimensions of polymer materials that ended their life cycle?

**Figure 9:**
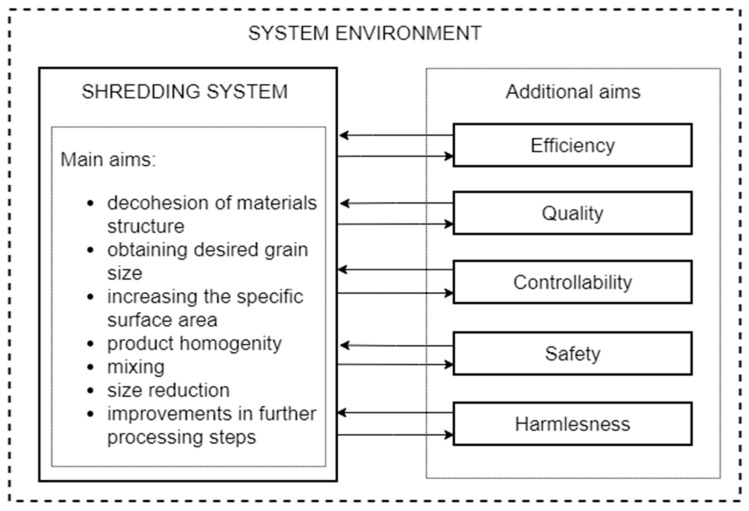
Objectives of the polymer materials shredding process in recycling. Own study based on [45,95,96,97].

The purposes of shredding in the context of EoL management are closely related to further use, the chosen path of development, and further processing [45,95,96,97]. J. Flizikowski in [45] distinguishes between the main and additional goals (Figure 9).

The most important goal is to reduce the dimensions to facilitate the following, among others: separation of materials, transport by conveyors, pneumatic transport, mixing of ingredients, as well as reducing transport costs and the storage area due to the reduction of waste volume [45,48,95,96,97].

With regard to landfill processes, shredding can be done to reduce the volume of waste, to clean up and stabilize the landfill, and to protect the bottom seals of the landfill. Usually, self-propelled machines are used to crush and break polymer materials into fairly large particles [48].

In energy recovery processes in the context of incineration, shredding is carried out in order to reduce the content of flammable substances in the residues, to improve transport and dose to and from the combustion chamber, and to facilitate mixing processes. The material sizes are reduced to dimensions in the range of 300–500 mm by means of various types of mills, e.g., rotary shears, hook shredders. Depending on the needs of the incineration plants, from 10 up to 100% of the prepared charge is reduced in size [48].

In the combustion of Refuse-Derived Fuel (RDF), the materials are shredded for the needs of fluidized beds and materials transport. Wastes are crushed into particles with dimensions from approximately 50 to 100 mm, although steelworks require fragmentation of the charge at a level of 15 mm, and cement kilns require one below 10 mm. Shredding usually takes place in two stages: the first stage is pre-shredding with e.g., rotary shears and hook shredders, and the second stage is secondary shredding to smaller particles with hammer mills, rotary shears, or impact mills [48].

In the case of shredding for recycling, the aim is in itself to reduce the form and dimensions and to facilitate the separation of different materials. The size of the particles after grinding depends on the way the material is used in recycling, e.g., whether the material will be used as a filler or the regranulate will be made of it, etc. In general, recycling uses a full range of grinding devices; very often, these are devices dedicated to the grinding of a given material [48].

## 3. Development of Shredding in Recycling

The basis for the achievement of the goals and development of shredding processes in recycling, as presented in [98], are as follows:

- Creative action (creation) understood as basics of shredding, creation of knowledge, resulting from system development, optimization, modernization, and innovation,
- Optimal parameters/processes/products—coming into possession of the process, machine (construction) design, or system condition taking into account the criteria enabling a rational assessment of condition,
- Modernization—intentional actions undertaken on the level of the technical system and the border zone; these actions aim to reduce the harmfulness of technology in the wider aspect considering improvement, restoration, and strengthening the environment properties.

The innovation should be carried out in a controlled manner, methodically, based on a specific mathematical model, which leads to revelation and revolution in the existing knowledge.

### 3.1. Types of Grinding

The shredding process consists of reducing the geometrical dimensions of the material (division, disintegration into smaller particles) as a result of the applied load (forces) induced by the movement of the shredding elements, causing stresses that exceed the strength (compressive strength, shear strength, and surface pressures) depending on the applied loads, thereby destroying and disintegrating material into smaller fractions. Depending on the nature of the applied loads and the stresses arising in the material, the following shredding methods are distinguished: crushing, shearing, abrasion, hitting, breaking (Table 2) [36,99,100,101].

The development of shredders for polymers disturbed during the use phase aims to obtain a product with a functional structure and the desired external form. Taking into account the grain size classes of the obtained product, shredding can be divided into coarse, medium, fine, and colloidal [46]. Table 3 shows the particle size range for a given type of shredding. The desired grain size range of the shredding product should contain at least 75% of the total mass of obtained material reduced in size [46].

**Figure 10:**
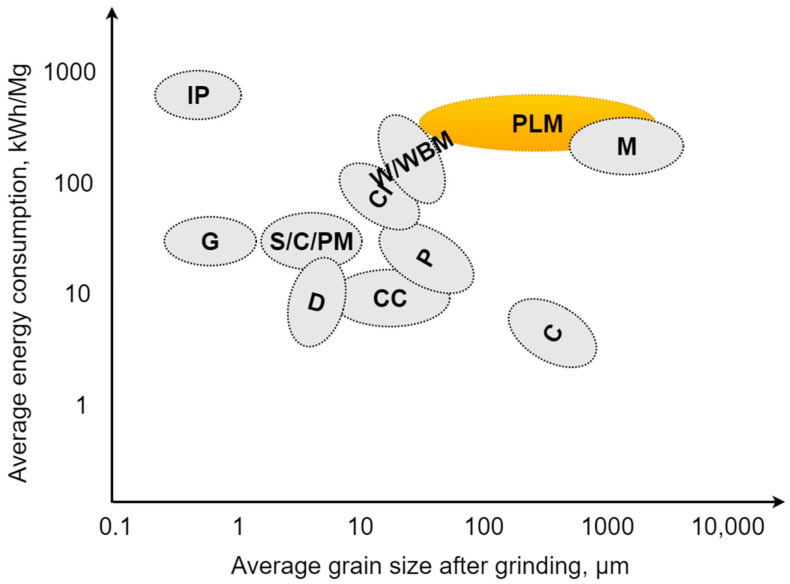
Variability of energy demand during the shredding of selected materials: IP—inorganic pigments, G—glass, D—dolomite, S/C/PM—sugar, cocoa, powdered milk, CC—cement, clinker, P—pepper, CI—cinnamon, W/WBM—wood and wood-based materials, C—coal, PLM—polymer materials, M—metals. Adopted from [36].

Obtaining a product with specific, desired dimensions required in further processing steps, e.g., combustion, production of fillers and regranulates, determines the choice of technology and appropriate parameters of the shredding process. Many designs of shredding machines and shredding units were created due to the post-use disturbances and the structural diversity of shredded polymer materials (from elastomers to more brittle and hard thermosets), for example: hammer, knife, disc, drum shredders, and many other modifications of the mentioned design solutions [102,103,104]. The use of a given type of shredder depends on the properties and contamination of the comminuted charge, the required particles size reduction ratio, the efficiency, the technical requirements, and the energy consumption per unit mass of the charge [45,105]. Figure 10 shows the ranges of energy demand during the comminution of selected materials. Polymer materials, marked in yellow in Figure 10, are a group of materials with high energy consumption needed for grinding (even more than 10 times greater than in the case of brittle materials). In case of polymers, particles after grinding usually do not reach dimensions less than 10 μm (often, particles do not even reach dimensions less than 1 mm) [36,106].

### 3.2. Quasi-Cutting Phenomenon

The material division, in the case of contaminated polymer materials, occurs as a result of the action of shearing forces and stresses (there is a complex state of stresses, including compression, shear, bending, with the dominant component being shear stress) caused in the material by two opposing edges moving relative to each other [36,107,108]. Due to the complex nature of the loads and the existence of a gap between the cutting edges (the cutting forces are not in one plane, they are shifted to each other), this type of shear is called quasi-technological [36,45,109]. It is mainly used for shearing plastomers and cross-linked elastomers [36,110]. The occurring phenomena are difficult to describe with detailed models, both on the material side and on the machine side (in terms of the friction models, temperature changes, energy, etc.); therefore, research and attempts are constantly being made to determine the relationship for this type of process, e.g., in studies [111,112,113].

**Figure 11:**
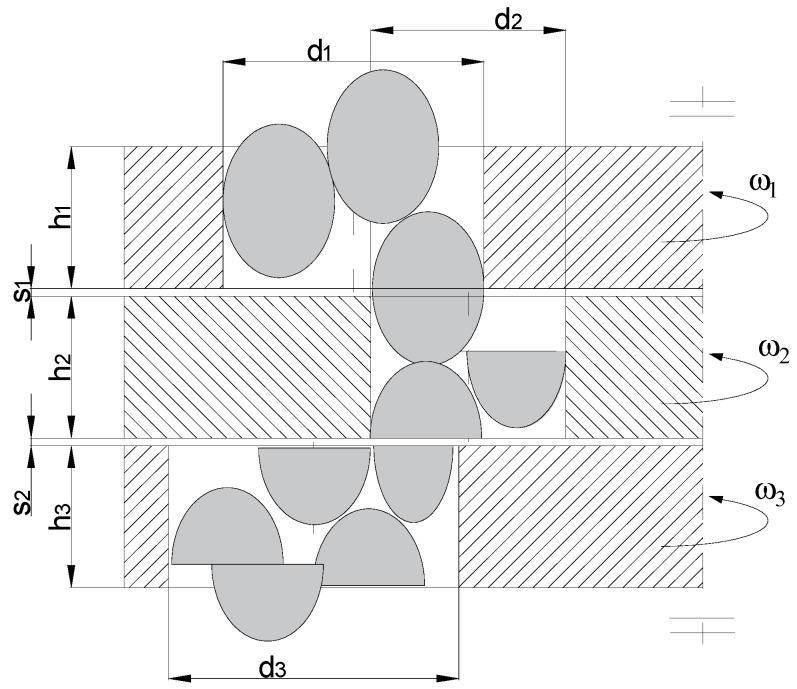
Model of quasi-cutting, d_1_, d_2_, d_3_—holes diameters, h_1_, h_2_, h_3_—disc height, s_1_—inter-disc gap, F—force, ω_1_, ω_2_, ω_3_—discs angular velocities [106].

The process of material passing through the shredding unit is characteristic in the case of quasi-cutting implemented among others, in drum shredders and in multi-hole, multi-disc shredders. The material entering the space between the cooperating cutting elements rotating with variable rotational speed is sheared as a result of the contact with cutting edges, as shown in Figure 11.

The condition must be met to shear the material between discs in a multi-hole disc unit [114]:

(7) $$\omega_{n-1}\neq \omega_{n}\;\mathrm{and}\;\omega_{n}\neq \omega_{n+1}$$

where *ω_n_*—angular velocity of the *n*-th shredding element, *ω_n−_*_1_—angular velocity of the preceding shredding element, *ω_n+_*_1_—angular velocity of the following shredding element.

### 3.3. Factors Affecting the Quasi-Cutting

**Figure 12:**
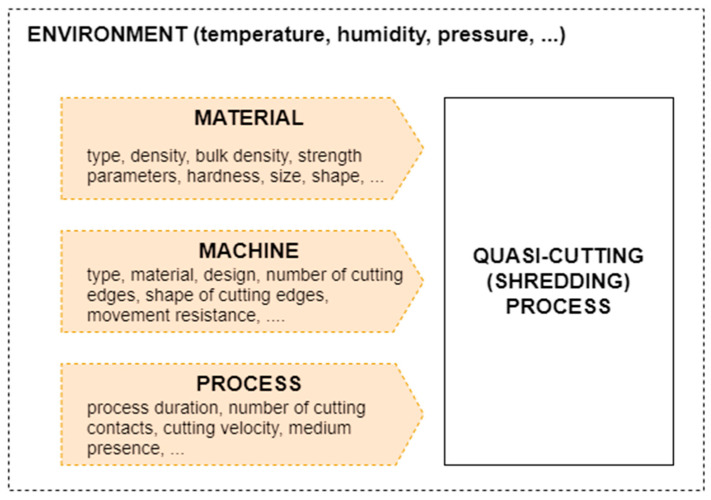
Factors influencing the quasi-cutting process. Own work on the basis of data from [115].

The parameters of the technological quasi-cutting process are influenced by many variable factors, ranging from the properties of the material to the operating parameters of the shredding unit (Figure 12) [115,116,117,118]. The knowledge of the variables influencing the quasi-cutting process is important from the point of view of the optimal process design [119]. These factors can be classified as material, machine, and process factors.

#### 3.3.1. Material Factors and Disturbances *d*(*m*)

Due to the loads, cutting is the most common process in the shredding of disturbed polymeric materials, so the mechanical properties related to cutting processes will be the most important from the point of view of the mechanical processing of polymers in recycling.

Some materials properties affecting energy-related shredding process indicators can be indicated considering polymeric materials recycling. They include the following [47,109,120,121,122,123,124,125,126]:

- Total energy of fracture propagation,
- Crack (cutting) stress,
- Crack (cutting) resistance,
- Load during collision and cutting,
- Collision duration,
- Performance ratio of ground product incineration,
- Relation of dimensions before and after the shredding process,
- Increase of specific surface area.

The strength properties and unevenness of loads are very important from the point of view of the mechanical processing and energy assessment of shredding processes [6,10,127]. The strength tests (for example: tensile, bending, shear test) are applied to a variety of polymeric materials with different properties, for instance: rigid thermosetting plastics for forming, filled and reinforced compositions, rigid thermosetting plates, laminates, thermosetting and fiber-reinforced thermoplastic composites (mats, chopped fibers, rovings, fabrics, composite, and hybrid reinforcements) [124,127].

Depending on the purpose for which they are used, they may exhibit poor or medium mechanical resistance [6,109,124]. This is crucial not only from the perspective of the preparation of the material but also from the perspective of its processing, use, and operation [17,36].

For energy balances and the efficiency of disturbed *d(m)* material disintegration, a very important parameter is cutting resistance, which describes the cuttability of the material. Generally, the less force applied to the material division, the greater the susceptibility to shredding it shows. The shredding unit design selection as well as the power and energy consumption for this process depend primarily on the materials’ cuttability [103,128]. As a rule, the higher the force values necessary for the material division, the higher the energy demand in the comminution processes [36,106].

**Figure 13:**
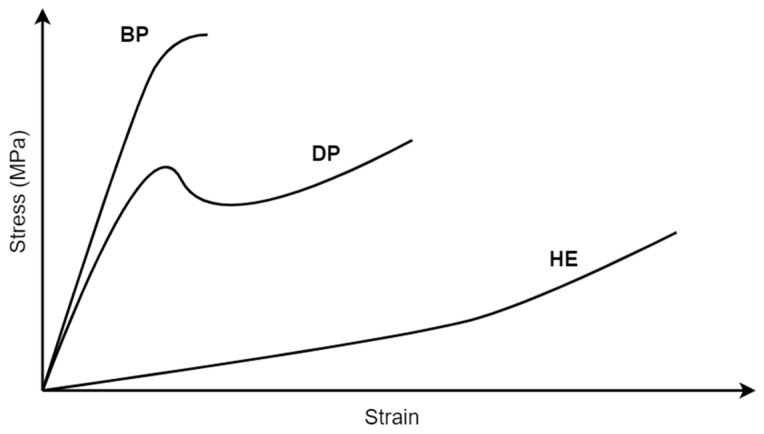
Behavior of different types of polymeric materials subjected to loads according to [126], BP—brittle polymer (glassy polymer, low-temperature thermoset), DP—ductile polymer (semi-crystalline, plastic, high-temperature thermoplastic, HE—highly elastic (elastomers).

The value of cutting resistance depends on two aspects: the intrinsic strength of the material and the occurring friction phenomenon [52,129]. The strength of the polymeric materials is affected by the type of the material, its molecular weight (the lower the molecular weight, the lower the strength), cross-linking (increases strength), and crystallinity (its increase causes an increase in strength) [126]. The strength is higher for brittle polymers (for instance, polystyrene) and lower for elastomers (rubber-like ones) [130] (see Figure 13). This diversification in the strength and properties of polymeric materials makes recycling and shredding of plastic waste problematic without the prior separation and segregation of waste by material type.

**Figure 14:**
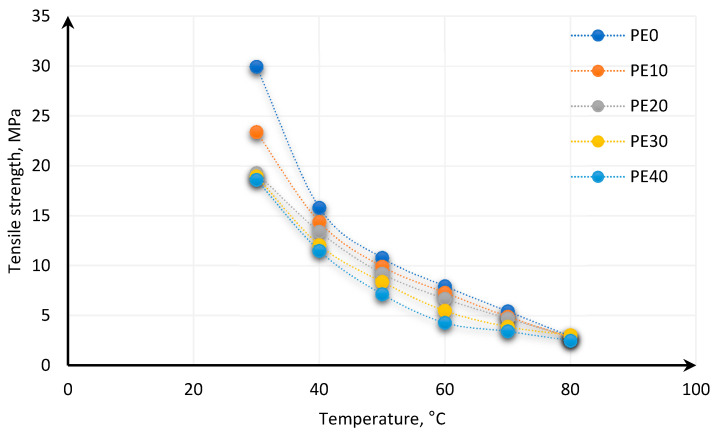
Dependence of tensile strength on disturbed temperature *d(T)* for different shares of Polyethylene (PE) in butyl rubber-based carbon black-reinforced elastomers. Graph created based on data shown in [131].

The strength of polymers is changing with the disturbed temperature *d(T)* due to changes in the internal structure caused by a temperature decrease or increase [126,131]. Hussein et al. [131] show the dependence of strength parameters of carbon black-reinforced elastomers based on butyl rubber and high molecular weight Polyethylene (PE) on temperature. The different share of PE in elastomer for different temperatures was tested. It was shown that the strength of the polymeric material decreases with temperature increase (Figure 14). Another conclusion from this study [131] concerned brittleness at critical temperature (*T_c_*). For all tested mixtures of butyl rubber and PE at temperatures above 60 °C, the typical brittle fracture was observed.

The second mentioned factor affecting cutting resistance is disturbed friction contribution *d(f)*. In [52], it was found that the increase in the friction coefficient can enhance or reduce the cut resistance. The energy consumption for cutting results in energy losses for dissipation and cutting energy at the edge of the blade. In this case, every increase in energy dissipation caused by friction increases the cutting resistance, while friction increase on the edges of blades reduces the energy requirements [52].

In another study [129], the cutting resistance of glassy and soft polymers was tested. It was also shown that the cutting resistance depends on the type of the material and its mechanical properties as well as cutting tool sharpness. It was found that the response of the material for cutting differs depending on the sharpness, while sharpness is affected by the geometry of the cutting blade and fracture toughness and the rigidity of the material. The toughness of a material is given by the area under a stress–strain curve and describes the energy absorbed by the material before fracture.

Millar et al. also investigated the cutting resistance [132]. They compared the fracture toughness of a polyester film and three polyester laminates obtained in cutting and tearing tests. This study shows that similar results of fracture toughness properties for the polyester laminates can be obtained both in cutting and tearing tests. They also found that the greater fracture toughness values of the polyester film were measured in tearing tests than in cutting tests.

#### 3.3.2. Factors and Disturbances *d*(*mp*) in Relation to the Grinding Machine and the Grinding Process

The factors and disturbances related to the machine operation and its design features, as well as some factors and disturbances affecting the grinding process, which influences the efficiency and size reduction ratio of the product during quasi-cutting, were indicated in [111] on the basis of research on a five-disc shredder. They include the following, among others [111]:

- The rake angle of the cutting edge on the material—it affects, inter alia, the size reduction ratio and energy consumption,
- The way of feeding the batch material to the shredding chamber (gravitational, forced)—it primarily affects the efficiency of the process; forcing the feeding speed may increase or decrease the efficiency depending on the relationship between the feeding speed and the processing capacity of the machine,
- The number of times the material is shredded and the number of contacts between the material and the cutting edges—increasing the number of contacts between the cutting edges and the material increases the particles size reduction ratio; unfortunately, it also increases energy demand,
- Geometry of the cutting edges and design of the entire cutting unit—primarily affects the efficiency and size reduction ratio,
- The material flow index for adjacent pairs of the cutting holes edges affects the efficiency; poorly selected relations of the hole sizes in the cutting discs may cause the material throttling and lower efficiency due to disturbances in the flow of particles in the shredding chamber,
- The size of the working gap between the cooperating edges primarily determines the size reduction ratio: the smaller the gap, the smaller the particles that can be obtained.

### 3.4. Quasi-Cutting Research of Contaminated Polymer Materials

The strength parameters under quasi-cutting test conditions in a quasi-static regime were obtained in [133] (velocity range 2–50 mm·s^−1^) and for polyester films and laminates in [132], which of course are much different than for dynamic conditions [49,134].

In papers [132,135], the shear phenomenon was studied along with the energy expenditure for cutting elastic–plastic polymers subjected to tensile pre-stress. The cutting energy per unit area of increased fracture surface in this case is defined as the sum of tearing energy T resulting from pre-stretching and blade interaction energy F, which can be expressed as [132,135]:

(8) G=T+F.

Then, tearing energy is determined from Relation (9), and the energy associated with the force applied to the blade is described by Equation (10). For pure shearing, it is assumed that *G* = *F*. The cutting energy determined based only on the blade cutting component for polyester laminates 3 M 9733, 3 M 9736, and 3 M 9730, was 1.8, 3.8, and 2.4 kJ/m^2^, respectively [132].

(9) $$T=Wh=\frac{\sigma^{2}h}{2E}$$

(10) $$F=\frac{f}{t}$$

where *W*—strain energy density, *h*—width of specimen without pre-stress, *σ*—stress, *E*—Young’s modulus, *f*—blade force, and *t*—thickness of specimen without pre-stress. The advantage of the determination method of the cutting energy described above is that the results are independent of the shape and size of the cut specimen, suggesting that they depend only on the material properties and sharpness of the knife [135]. In [133], a method of determining the quasi-cutting forces’ quasi-static regime was proposed. The special construction multi-disc, multi-hole apparatus for cutting [136] was used toward the cognition of processing behavior of PE and PVC tubular post-use pipes. The design solution given as a logical conjunction of criteria with possible—from the area of the common design features in the conceptual space, simulation, strength, and structural-processing tests (suboptimal) can be described on the basis of the identified relation between forces, cutting blade angle, and displacement for contaminated polymer materials. The initial behavior of polymers subjected to stress (for e.g., in recycling processes) and class of the process were described. The parameters, test conditions, and results for different materials and blade angles are shown in Table 4.

The strength tests, described in [133], show that the mean strength of the Low-density polyethylene (LDPE) post-use pipe is *R_t_* = 11.97 MPa, standard deviation *s_R_* = 1.5114; (*d* = 11.66–44.48%). The investigation results suggest principles to show the selection of settlement regarding the most suitable method of disintegration and estimation of work and loads. In addition, discerning the analysis of sub-ranges of the chart (in Table 4) gives aids to selection of the optimal way of disintegration. However, the general solution for every chart (Table 4) is described by 4th degree equation assigned area of possible search [133]:

(11) $$P=a\Delta l^{4}+b\Delta l^{3}-c\Delta l^{2}-d\Delta l+e\;\mathrm{for}\;R^{2}>0.95.$$

The force models of polymeric pipes shredding in dependence of displacement were established on the basis of the strength test [133]:

(12) $$P=-10^{-5}\Delta l^{4}-0.0005\Delta l^{3}+0.1311\Delta l^{2}-5.3225\Delta l+63.778\;\mathrm{for}\;R^{2}>0.9144$$

(13) $$P=-3\cdot 10^{-5}\Delta l^{4}+0.003\Delta l^{3}-0.1053\Delta l^{2}+1.6588\Delta l-10.598\;\mathrm{for}\;R^{2}>0.932$$

(14) $$P=-4\cdot 10^{-7}\Delta l^{4}-5\cdot 10^{-6}\Delta l^{3}-0.0103\Delta l^{2}+0.7973\Delta l-10.824\;\mathrm{for}\;R^{2}>0.8514$$

(15) $$P=-5\cdot 10^{-5}\Delta l^{4}+0.0058\Delta l^{3}-0.258\Delta l^{2}+4.8103\Delta l-26.105\;\mathrm{for}\;R^{2}>0.8824$$

In addition to the assessment of strength under a quasi-static regime, tests were carried out on a single shredding process under dynamic conditions [110,133,137,138,139,140,141,142]. Such tests provide knowledge of the idealized process of shredding the sample together with a preliminary assessment of the type of load occurring in the sample. The dynamic test allows determining the impact resistance—also one of the most important mechanical properties of polymeric materials [110,137,141]. Impact resistance is understood as a measure of the fragility of materials. It is expressed by the ratio of the force used for the dynamic fracture of the specimen. The impact strength refers to the cross-sectional size of the specimen. The unit in which it is expressed is kJ·m^−2^.

The impact strength of polymers materials can be determined via a Charpy impact test. This test is suitable for a wide range of plastics: from brittle thermosets to high-impact polymer blends [137]. The use of a traditional Charpy hammer in this study allows for the observation and analysis of specimen fracture under single-impact hammer contact conditions, but it does not capture the nature of dynamic cutting that occurs in multi-disc shredders [141].

In order to simulate the dynamic shear conditions, a modified research stand based on the Charpy method in the study of disturbed polymer materials impact resistance was proposed in the paper [133]. The principle of operation of the stand is based on dynamic impact of the edge of the tool—a hammer (equivalent to a shredder knife). In this research, a modification of the hammer was applied. A replaceable blade was attached to the end of the hammer, which caused dynamic shear when in contact with the specimen. The research presented in [133] was carried out to improve knowledge about the shredding process. It allowed estimating the amount of energy needed to shred the sample once. This requires the specification of appropriate parameters, such as the shape of the grinding tool and the speed of its impact on the sample. As a result of the impact load, the kinetic energy of the knife *W* = 0.5 *m* · *v*^2^ is converted into the deformation of the test sample. The work was described by a formula [133]:

(16) $$W=\frac{m\cdot v^{2}}{2}=\int_{0}^{F_{\max}}F\cdot df\approx \frac{F_{\max}\cdot f}{2}$$

where *m*—mass of the hammer, *v*—velocity of the hammer during collision with the specimen, *F*—force acting on the specimen, and *f*—deflection.

In [133], the tests were conducted using a modified Charpy hammer and three knife angles—60°, 75°, and 90°, and in [141], 70°, 80°, and 90° knife angles were tested. The recording of momentary loads in the single shredding test was carried out using the Spider 8 measuring system from Hottinger Baldwin Messtechnik. The course of sample destruction was additionally captured by a camera recording the image at a speed of 1200 frames per second. Samples used in the tests were 14mm x 8mm. The cross-section of each sample was 1.75 mm^2^ [133].

**Figure 15:**
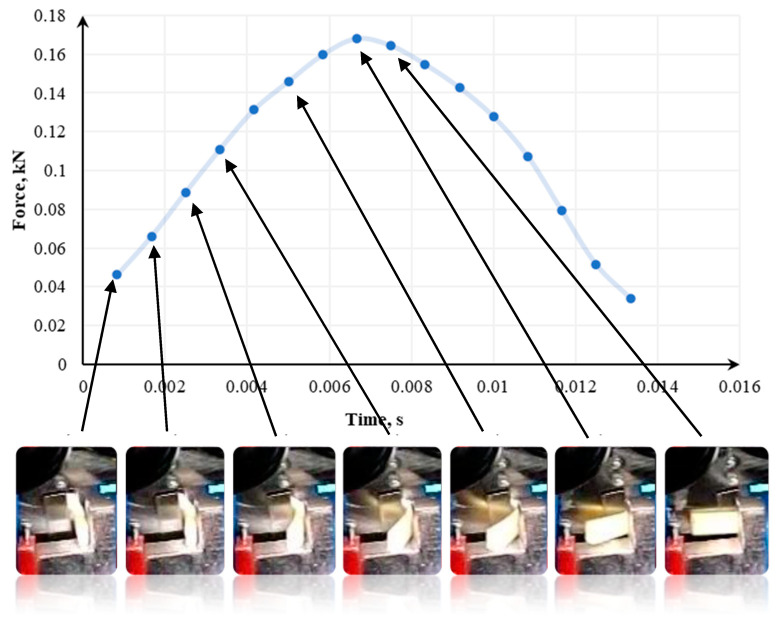
Momentary force values during grinding of a single polypropylene sample in a vertical arrangement using a 60° knife.

**Figure 16:**
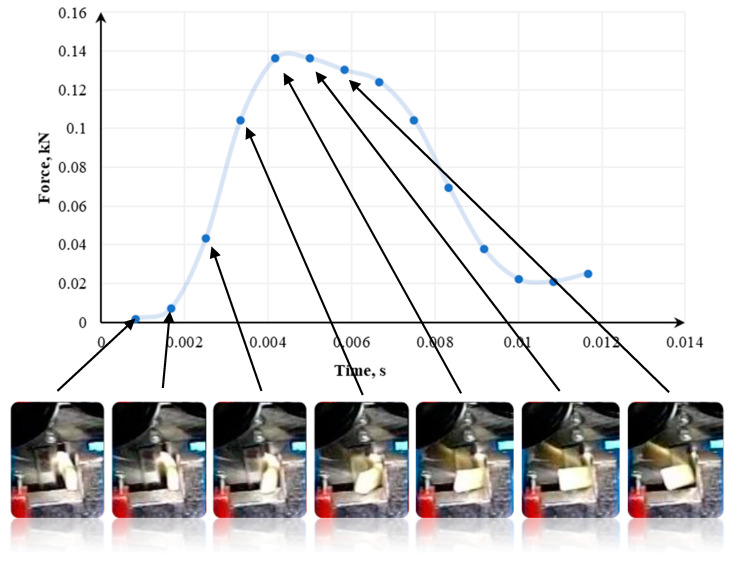
Momentary force values during grinding of a single polypropylene sample in a vertical arrangement using a 75° knife.

**Figure 17:**
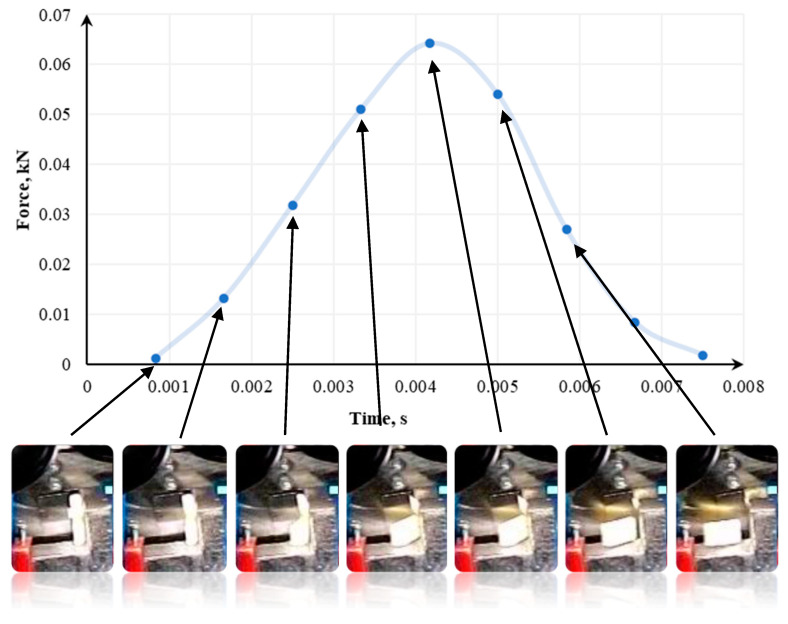
Momentary force values during grinding of a single polypropylene sample in a vertical arrangement using a 90° knife.

Figure 15, Figure 16 and Figure 17 show how the quasi-cutting of the polypropylene sample was carried out for different knife angles successively going deeper into the material during cutting. The experimental tests [133,141] as well as computer simulation results presented in [142] proved that the sample is deflected radially from the blade toward the outer edges of the sample. It was found that the polypropylene test specimen with a filler content of 1% (tensile test paddle) compressed and deformed locally at the point of impact. A piece of specimen based on a yoke on the side on which the blade operates is also compressed.

As shown in [110,133,141,142], in parallel to compression, a brittle breakthrough occurs perpendicular to the edge of the specimen. The crack appears about 1 mm from the area where the blade hits—in the plane of attack. There are also cases of micro-cracks spreading from the blade at an angle of about 82° [133,141]. Each time this type of situation occurs, a portion of the specimen between the base (yoke) and the blade application plane is cut. In research presented in [133,141,142], a crack occurs between the blade and the edge of the support. However, there is no complete breakthrough. Part of the sample is torn off, and the residue is supported by the plane of attack. One of the semi-circular components is disintegrated on impact. For a blade angle of 90°, the entire surface of the plane of attack can be observed. The decay of the material is uncontrolled for this test. In the case of a knife whose blade reached an angle of 90°, not only a crack in the middle part of the sample (without a breakthrough) occurs. In addition, it rotates and slips between the blades. Under the conditions of energy tests, no breakthrough was shown when the sample was horizontal. In other cases, it was optimally disintegrated.

The influence of the blade angle on the cutting process and the strain energy was also studied in [110,140]. In contrast to the studies presented in [133,141,142], cutting of polymeric materials was performed on a specially designed granulator-like cutting test bench [110,140]. The material is sheared by a rotor knife, which is attached to the rotor. The rotor knife moves with a peripheral speed v around a circle with a radius equal to the length of the rotor arm. Then, the specimen is sheared by two opposite edges: the edge of the stator on which the specimen is placed and the edge of the rotor knife blade, which are face-to-face distances from each other by a certain distance s. The material splitting occurs as a result of the cutting load and shearing load. Cutting tests of polypropylene specimens for blade angles of 30°, 55°, and 80° indicated that an increase in blade angle results in an increase in cutting energy, which is due to a longer cutting distance until the specimen breaks for larger blade angles and higher cutting force values occurring [110,140]. It was also found that a larger portion of the cutting energy is used for friction and deformation processes.

Then, we confirm the complexity of processes affecting the shredding of polymers. The decisive factor in the course of decohesion and the amount of energy demand is the blade angle of the knife. If the sample is made of polypropylene with a 1% filler content, the energy requirement varies, depending on the angle of the blade. This is confirmed by observations of the course of the phenomenon, or more precisely, by optimally directed stresses, which appeared at a blade angle of 75° [133,141].

Based on quasi-shear tests in single impact tests, it is possible to draw conclusions about the nature of the materials structure and the cutting process. Certainly, these conclusions will be different for each material, because the parameters will be different. It is important to capture the course of the test in detail, because only then can the material variant be selected (looking through the prism of minimizing energy demand). In theory complemented by practice, we can see that the Charpy hammer is characterized by an uncomplicated construction, but it is worth considering its modifications—as in the case of the quoted research. It may allow obtaining smaller deviations in particular measurements, which translates into their accuracy.

### 3.5. Research in Machine (Shredder) Conditions

Basically, in the search for the appropriate constructional solutions of shredding equipment, the issues of usefulness of the unit shredding method are taken into account. This method makes it possible to estimate the best shredding condition in terms of geometrical features, loads, and speed of the working element [49,51]. Taking into account environmental appraisals for the search for more friendly technologies for materials shredding in recycling process, research is carried out to reduce the energy demand for this processes [143,144,145].

#### 3.5.1. Quasi-Cutting Machines

**Figure 18:**
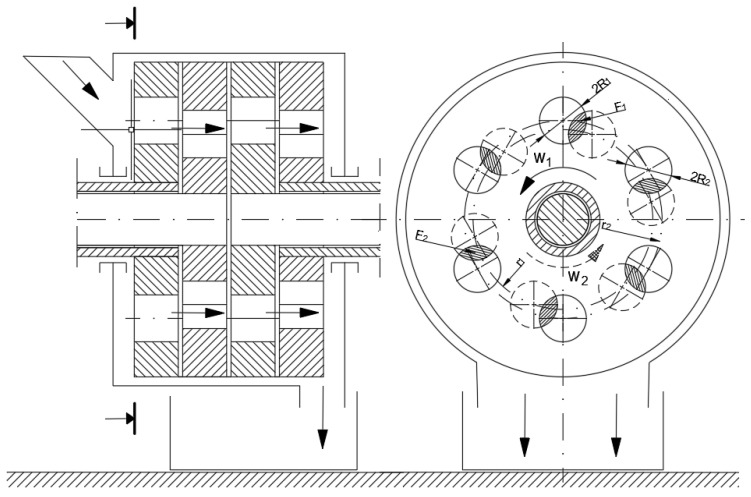
Diagram of the material flow in a multi-disc grinder with determination of the effective grinding areas [106].

Considerations of the quasi-cutting process in the machine conditions will be discussed regarding the example of multi-hole, multi-disc grinders. Understanding the relationship between the disc movement and mass flow is a crucial element to understand the essence of the multi-disc grinding process and the phenomena occurring in it, as they have a significant impact on the efficiency and effectiveness of disturbed polymer materials grinding. Figure 18 shows a diagram of the material flow through a disc, multi-hole grinding unit.

The mutual movement of the crushed disturbed polymeric material and the crusher’s structural elements was called the motional characteristics [70,71,98,146,147,148]. Two states of motion appear during EoL polymeric elements shredding [71,148,149]: idle state—material is not shredded, it is mixed and moved in the shredding chamber (for multi-hole cutting assembly, it appears for a linear velocity of cutting edges below 0.7 m·s^−1^) and shredding/working state—the appropriate shredding process takes place, the material breaks down into smaller particles [50,114,141,149,150,151].

Research conducted so far under quasi-cutting in machine conditions concerned the impact of multi-disc shredder design on the product quality, properties of the charge, grinding efficiency, and the relation between material properties and grinding energy [114,152,153,154,155].

The energy relations of multi-disc grinding have been described many times in the studies of Flizikowski [111,156,157,158]. His considerations are focused on innovative modeling of grinding process energy and environmental relations, intelligent development of the multi-disc grinding units design with the use of advanced computer techniques in the disc design process, and grinding process self-regulation [25,35,70,159,160].

The relationships between the material flow and the charge angle of repose on the grinding efficiency in a five-disc mill were discussed by A. Tomporowski in the studies of [149,150] and M. Opielak [150,161]. They also dealt with the issues of uneven grinding [149,162,163]. Table 5 presents examples of research areas on the multi-disc grinding process in the last 25 years.

The research on the comminution process discussed so far indicates that the environmental and economic aspects of comminuting disturbed polymer materials, as well as the grinding phenomena modeling, are not discussed or treated too vaguely.

#### 3.5.2. Shredding Area

The grinding cross-section *F_r_* (see Figure 18) was determined on the basis of the research and experiments for the multi-hole, multi-disc grinder design, and it is described by the following relationship [36]:

(17) $$2F_{r}=\frac{6.11}{\rho \cdot s_{i}}\left({\left(0.043\cdot s_{i}\right)}^{-1.12D_{oi}+1.18}\right)\cdot v_{r}\cdot e^{1.88n_{w}^{2}}$$

where *F_r_*—grinding cross-section, mm^2^, *ρ*—shredded material density, g/cm^3^, *s_i_*—the index of the gap between disks (drums) of the grinder, (-), *D_oi_*—diameter indicator of the *i*-th disk (drum) provided with holes, (-), *n_w_*—the factor dependent on the type of processed materials; *n* = 0.75÷1.00, *v_r_*—relative speed between quasi-cutting edges, m∙s^−1^.

The grinding cross-section is crucial for the grinding efficiency. The larger the cross-section, the greater the efficiency, because more material flows through the grinding chamber. Unfortunately, it is associated with an increase in energy consumption [36,107,111].

In the research conducted by Sadkiewicz and Flizikowski [111], it was shown that during the materials grinding in multi-hole, multi-disc grinding units, it may happen that the entire cross-section of holes is not used, which is related to the different speeds set on the discs. Only a certain part of the hole area (grinding cross-section) is effectively used. For pairs of holes in adjacent shields, the following relationship was determined [111]:

(18) $$K_{\left(i+1\right)/i,j}=\frac{F_{i+1}}{F_{c}}$$

*k*_(*i*+1)/*i*,*j*_—the ratio of the effective area of the material flow between the pairs of holes of adjacent discs, *F*_*i*+1_—hole total area in the following shredding disc, m^2^, *F_i_*—hole total area of the preceding shredding disc, m^2^.

The total area for matching holes of adjacent discs can be determined from the formula [111]:

(19) $$k_{n\left(i+1\right)k\left(i,j\right)}=\frac{\sum F_{n\left(i+1\right)}}{F_{k\left(i\right)}}$$

*K*_*n*(*i*+1)*k*(*i*,*j*)_—material flow ratio for the total area of the holes of adjacent discs, Ʃ*F*_*n*(*i*+1)_—total area of holes in the next shredding disc, m^2^, *F_ki_*—total area of holes in the preceding shredding disc, m^2^.

An estimator of the standard deviation of the comminution cross-section was proposed to assess the operation stability and implementation in the disturbed polymer materials comminution control systems [107]:

(20) $$\Delta F_{r}=\sqrt{\frac{n\cdot \sum_{i}F_{r,j}^{2}-{\left(\sum_{i}F_{r,j}\right)}^{2}}{n\left(n-1\right)}}.$$

The use of the standard deviation allows reading the suitability of the design in terms of the machine stable operation, and at the same time, small deviations from the average guarantee the low variability of energy demand [107].

#### 3.5.3. Quasi-Cutting Process Indicators

**Figure 19:**
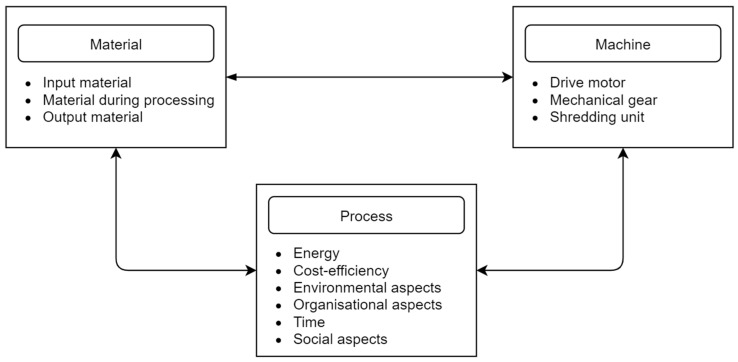
The objects in shredding processes in disturbed polymer material recycling and their relations.

In the previous studies [169,170,171,172], many indicators of shredding processes, for instance efficiency, production yield, unit energy consumption, and product quality, have been described and analyzed. To determine the values of mentioned indicators, some specific tests and experiments should be conducted. In shredding processes in disturbed polymeric material recycling, the following groups of objects can be distinguished (Figure 19) [98]:

- Material,
- Machine,
- Process.

The material indicators can be considered for input material, material during processing, and output material. The machine as an object is described by the indicators and parameters of the drive unit, gears, and shredding unit (especially its design and movement parameters). The shredding process can be described by many indicators, but the most important are related to energy, efficiency (including cost-efficiency), environmental, social, and organizational aspects and time. It is necessary to require detailed knowledge on the properties of specific groups of objects: the material, the functional unit, and the shredding process itself [98].

The grinding process indicators include many aspects, e.g., effectiveness, product quality, process quality, efficiency, energy efficiency, costs, etc. The classification of grinding indicators was done by Macko in [173]. He distinguished technological, technical, and economic indicators (Table 6); however, environmental impacts of shredding were not included in this classification. These, in turn, were included in the classification made by Flizikowski [45,174], who classified the grinding process indicators into three groups: quality, efficiency, and harmlessness (Table 7), which significantly extends the range of indicators important for grinding. In another study [106], the following indicators groups were proposed:

- Product (properties such as particle size distribution, specific surface area, bulk density, viscosity, etc.),
- Process (energy, technology, quality),
- Shredding environment (generated noise, emissions of solid, liquid and gaseous waste, generated vibrations, etc.).

The research carried out so far describes many experimental and theoretical works, including theories about shredding processes. The most popular are energy indicators, including theories by Griffith [175], Behrens [176], Rumpf [177,178], Schonert [179,180,181,182,183], Kerlin [184], and Flizikowski [45,98,156]. One of the simplest indicators for the global evaluation of the shredding process in the machine conditions is the unit energy consumption indicator *E_j_*, which specifies the amount of electricity needed for grinding the mass unit of the material [97]:

(21) $$E_{spec}=\frac{1}{m}\int_{t_{0}}^{t}\left(P_{T}-P_{l}\right)dt$$

where

*E_spec_*—specific energy consumption, J·kg^−1^,

*P_T_*—total power consumption during grinding, W, and

*P_l_*—power consumption for idle run, W.

The decrease in the specific energy consumption is the effect of increased efficiency and energy efficiency of the grinding process. Since the purpose of comminution is to obtain a product with specific required dimensions, the measure of process and product usefulness may be the target energy consumption index *E_Rq_* expressing the amount of energy for comminution related to the efficiency of obtaining a product with specific dimensions [185]:

(22) $$E_{Rq}=\frac{P_{r}}{W_{f_{q}}}$$

where

*P_r_*—power consumed for grinding process [kW],
*W_fq_*—efficiency of obtaining material with desired dimensions [kg∙h^−1^].

The analysis of the research carried out so far shows that the specific energy consumption depends on the rotational, linear speed of cutting elements, and it increases with increasing speed. The demand for energy also depends on the material type and its strength properties [1,32,44,106,170,186,187], as well as on the machine design features, which affect the demand for power and efficiency [36,149,150]. For example, a lower power consumption was found for a multi-disc, multi-hole shredder with polygonal holes than for a shredder with discs with round holes [36].

Another important parameter for evaluating the comminution process is efficiency. It is determined by disturbances *d*(*m*), *d*(*f*), *d*(*T*), *d*(*mp*), etc. and by key process and design parameters. Efficiency (production yield) mainly depends on the holes volume in the first disc, disc rotational speed, and the dosing efficiency, which determines the level of filling the space of the grinding holes. Therefore, the mass efficiency of grinding *Q_r_* (g·s^−1^) can be described by the function [106]:

(23) $$Q_{r}=f\left(V_{otwT1},\omega_{1},\delta ,t\right)$$

where

*V_otwT_*_1_—volume of grains introduced into the holes in the first disc, m^3^,
*ω*_1_—angular velocity of the first disc, rad·s^−1^,
*δ*—hole filling factor, –,
*t*—time, s.

The mass efficiency in machine conditions can also be monitored on the basis of the dependence of the change in the grinding product mass Δ*m* in the receiving basket during the observation Δ*t* [1,32,106,170,186]:

(24) $$Q_{r}=\frac{\Delta m}{\Delta t}.$$

The efficiency depends, as in the case of unit energy demand, on the rotational speed of the cutting elements. As shown in previous studies, an increase in the grinding elements rotational speed causes an increase in efficiency [1,32,44,106,170,186,187]. As indicated earlier, the efficiency also depends on the design features of the shredding unit and in particular on the effectively used grinding area (cross-section), which in the case of multi-disc shredders is determined by the shape, number, and size of holes in the discs [36,107,111].

The size reduction ratio is the most frequently used indicator for the grinding effects evaluation. It can be determined in many ways, e.g., by means of the limit size reduction ratio *i_g_* [1,32,106,170,186]:

(25) $$i_{g}=\frac{D_{\max}}{d_{\max}}$$

where

*D*_max_—arithmetic mean of the diameters of the largest grains of the feed,
*d*_max_—arithmetic mean of the diameters of the largest grains of the grinding product.

The material size reduction ratio depends, among others, on the cutting elements’ relative velocity, the number of collisions with the cutting elements, the internal structure of the material, and the material strength properties. Increasing the speed of the cutting elements leads to an increase in the size reduction ratio, similarly as increasing the number of collisions with the cutting elements. As a rule, brittle materials will achieve higher values of size reduction ratio [1,32,44,106,170,186,187].

## 4. Recycling Potential in the Context of Shredding

On the basis of the principles of recycling development, the basics of shredding for the recycling of disturbed polymer materials can be formulated: the construction, process, equipment, systems, and machinery depend on human, technical, energy–material, and control potential. A comprehensive equation covering all novelties in operational shredding systems, from idea to elimination, has the following form [188]:

(26) $$L\left(\bar{H},\bar{E},\bar{R},\Theta ,t\right)=P\left(\bar{s},\bar{z},\Theta ,t-t_{0}\right)$$

where $\bar{H}$—performance characteristics as output quantities (efficiency), *Ē*—inner elements ((*nS*) new construction solutions) and outer elements (ready markets), $\bar{R}$—connections of elements (relations, reactions, correlations of elements),Θ, *t* − *t*_0_—time, $\bar{s}$—intentional control, $\bar{z}$—disturbances.

The performance characteristics, components and relationships of the polymer plastic recycling system are controlled, disrupted, time-variable, and dynamically created. According to designation, the functional plastics recycling for energy engineering spheres as a technical system is the whole of its external operating possibility and can be described by the function of operating potential [189]:

(27) $$P_{O}\left(t\right)=\Phi \left[P^{H}\left(t\right),P^{T}\left(t\right),P^{E}\left(t\right),P^{C}\left(t\right)\right]$$

where *P^H^(t)*—human potential, *P^T^(t)*—technical potential, *P^E^(t)*—energy–material (plastic) potential, *P^C^(t)*—controlling potential.

The following ones belong to indicators describing the operating potential (the description is limited to controlling potential exclusively, as the basic concept tool of designer’s activity):

- Current executive possibilities, *π_O_(t),*
- Volume of operation used actively, usefully *M_O_(t),*
- Theoretical possibilities and operational needs, *ε*,

and especially:

(28) $$P_{O}\left(t\right)=\pi_{O}\left(t\right)\cdot M_{O}\left(t\right)\cdot \varepsilon .$$

Taking into account the current state of knowledge, some simplifications and idealizations of the human, technical, energy–material, and control capabilities must be applied. The first idealization is the general equation of the potentials of action (27), and the second one is a detailed equation of the energy potentials balancing (28) in the recycling of polymer waste.

Operating (energy) potential equation in the period [*t*_0_,*T*] takes the following form [189]:

(29) $$P_{O}\left(T\right)=P_{O}\left(t_{0}\right)-\int_{t_{0}}^{T}p_{O}^{U}\left(t\right)dt-\int_{t_{0}}^{T}p_{O}^{S}\left(t\right)dt+\int_{t_{0}}^{T}p_{O}^{r}\left(t\right)dt$$

where *P_O_*(*t*_0_)—initial operating potential, $p_{O}^{U}(t)$—density of the effectively used stream of potential,$p_{O}^{S}(t)$—density of lost stream of potential,$p_{O}^{r}(t)$—density of recovered (or obtained from the environment) stream of potential.

Taking into account energetically plastics shredding for energy engineering aims, we obtain [189]:

(30) $$P_{em}\left(T\right)=P_{em}\left(t_{0}\right)-\int_{t_{0}}^{T}p_{em}^{U}\left(t\right)dt-\int_{t_{0}}^{T}p_{em}^{S}\left(t\right)dt+\int_{t_{0}}^{T}p_{em}^{r}\left(t\right)dt$$

where *P_em_*(*t*_0_)—initial energy–material potential (*e-m*) of the plastics shredding system, $p_{em}^{U}(t)$—flux density of effectively used *e-m* raw, plastics potential,$p_{em}^{S}(t)$—flux density of wasted and lost *e-m* plastics potential, $p_{em}^{r}(t)$—flux density of *e-m* recreated plastics potential (or only retrieved from environment).

### 4.1. Material and Technical Potentials (P^E^(t); P^T^(t))

The material *P^E^*(*t*) and technical potential *P^T^*(*t*) of disturbed polymer materials shredding are closely related to each other. Material potential *P^E^*(*t*) is understood as a resource of material that can be used in various forms, taking into account material properties, including processing properties, while the technical potential *P^T^*(*t*) is described as all means and methods enabling the use of the material potential *P^E^*(*t*). It is obvious that materials properties require the use of specific means and methods of processing, while the methods of processing will have an impact on the quality of the final product, processing efficiency (degree of processability—material recycling), and environmental effects.

The technical potential of the materials processing and recovery in recycling is determined by the equation [190]:

(31) $$P^{T}(t)=\frac{T^{T}\pi \left(t\right)}{M^{T}\left(t\right)\varepsilon^{T}}$$

where

*T^T^*—all technical potential intended for polymer materials recycling,
*M^T^*(*t*)—number of machines (shredders) taking part in recycling,
*ε^T^*—theoretical technical possibilities,
*π^T^*—real technical possibilities of machines,

and

(32) $$\varepsilon^{T}=\varepsilon_{k}^{T}\varepsilon_{p}^{T}\varepsilon_{e}^{T}\Rightarrow 1.$$

Ideally, the theoretical technical potential is 1. The conditions that must be met to obtain the maximum theoretical technical potential are as follows [190]:

- The construction with its substantial scope includes also the destruction, $\varepsilon_{k}^{T}$ = 1
- The building and machine are constructed according to the construction, $\varepsilon_{p}^{T}$ = 1
- Use, operation, resistance, and durability are adequate, $\varepsilon_{e}^{T}$ = 1.

In a similar way to the technical potential, one can build a dependence on the material potential [190]:

(33) $$P^{E}\left(t\right)=\frac{E^{E}\pi \left(t\right)}{M^{E}\left(t\right)\varepsilon^{E}}$$

where

*E^E^*—amount of useful energy and matter introduced into the system,
*M^E^*(*t*)—material and energy resources used in the processing,
*ε^E^*—theoretical material and energy possibilities,
*π^E^*—real, useful material and energy possibilities.

Achieving the maximum use of the theoretical energy–material potentials (*ε^E^* = 1) will be possible when [190]:

(34) $$\varepsilon^{E}=\varepsilon_{q}^{E}\varepsilon_{l}^{E}\varepsilon_{p}^{E}\varepsilon_{s}^{E}\varepsilon_{u}^{E}\Rightarrow 1$$

- Only renewable energy is used in processing, $\varepsilon_{q}^{E}$ = 1,
- The risk of depletion of water and food resources is minimized, $\varepsilon_{l}^{E}$ = 1,
- Processes are carried out in a sufficiently short time, $\varepsilon_{p}^{E}$ = 1,
- Maximum security of the processing is ensured, $\varepsilon_{s}^{E}$ = 1,
- Waste energy and waste (solid, liquid, gases) are minimized, $\varepsilon_{u}^{E}$ = 1.

### 4.2. Integrated Efficiency

**Figure 20:**
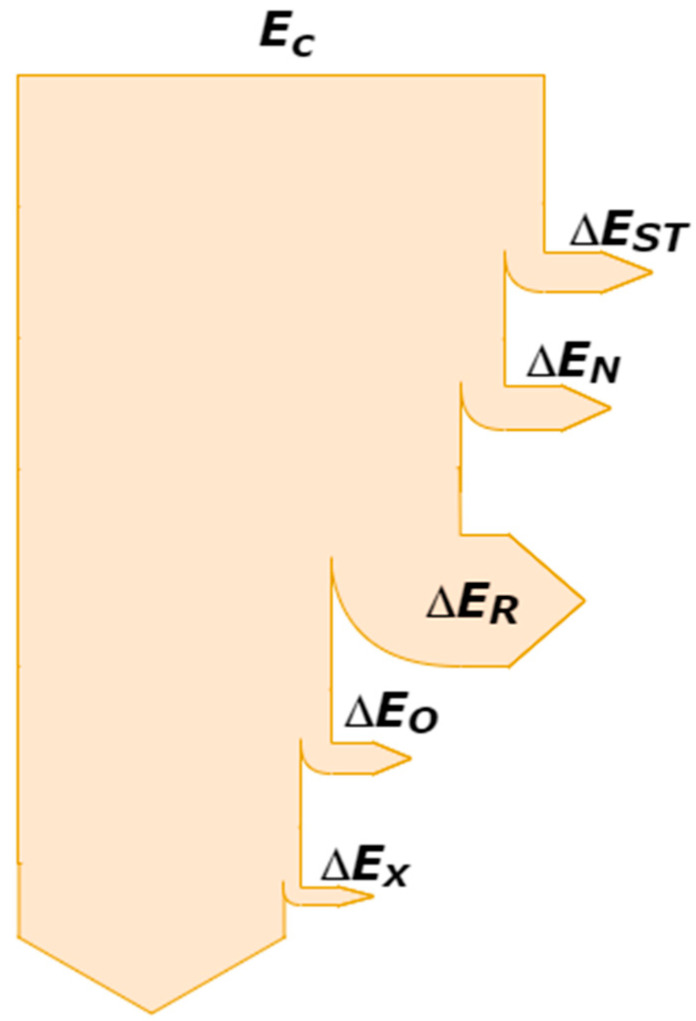
The energy flow during the shredding process.

The shredding system of disturbed polymeric materials is composed of many elements that affect the final energy balance and efficiency of the shredding process. The energy supplied to the system (drive system/motors) before it is effectively used for comminution is lost in the control system, in the drive train, in the grinding unit, in the raw material dosing system, or it is dispersed due to vibration (Figure 20) [35].

In [35], the energy balance model of the shredding process was proposed:

(35) $$E_{C}=\Delta E_{ST}+\Delta E_{N}+\Delta E_{K}+\Delta E_{O}+\Delta E_{X}$$

where *E_C_*—total energy supplied to system, Δ*E_ST_*—energy assigned for process control, Δ*E_N_*—energy lost in driving (motor and, gear) system, Δ*E_R_*—energy lost in cutting mill unit, Δ*E_O_*—energy assigned for process servicing, Δ*E_X_*—energy dissipated in system (vibrations, heat, sound, waves, etc.).

The energy potential is the most important factor for the shredding process related to shredding and driving assembly. From the point of view of design requirements and energy consumption problems, the relationship (30) was simplified [35]:

(36) $$E_{cR}=\int_{t_{0}}^{T}p_{d}^{E}\left(t\right)dt-\int_{t_{0}}^{T}p_{d}^{S}\left(t\right)dt\Rightarrow E_{C}=\Delta E_{N}+\Delta E_{R}\;\mathrm{or}\;E_{C}=E_{E}\;\mathrm{and}\;E_{R\lambda }=E_{T\lambda }+\Delta E_{M}$$

Assuming that *E_C_* = *E_E_* is electrical energy, the relation describing the energy necessary for the machine shredding of polymeric materials takes the following form [35]:

(37) $$E_{R}=\int_{t_{0}}^{T}p_{O}^{U}\left(t\right)dt\Rightarrow E_{R\lambda }=\int_{0}^{T}P_{R}\left(t\right)v\left(t\right)dt\;\mathrm{for}\;P_{R}>0,v>0$$

where *P_R_(t)*—shredding force, *N*, *v(t)*—shredding velocity, m·s^−1^, *E_Rλ_*—energy consumed to obtain the desired degree of fineness of plastics output material, *J*, *T*—time of grinding in one cycle, *s*.

Based on [35], it can also be written as follows:

(38) $$E_{R\lambda }=\int_{t_{0}}^{T}p_{O}^{U}\left(t\right)dt-\int_{t_{0}}^{T}p_{O}^{S}\left(t\right)dt\Rightarrow E_{R\lambda }=E_{E}\cdot \eta_{s}\cdot \eta_{p}\;\mathrm{or}\;E_{R\lambda }=E_{E}\cdot \eta_{n}$$

where *ƞ_s_*—engine mechanical efficiency, *ƞ_p_*—gear(s) efficiency, *ƞ_n_*—power drive efficiency, and *E_E_*—electric energy supplied to the engines, *J*.

For the stabilized motion of shredding cycles, it may be assumed that [35]:

(39) $$E_{R\lambda }=\int_{t_{0}}^{T}p_{O}^{U}\left(t\right)dt-\int_{t_{0}}^{T}p_{O}^{S}\left(t\right)dt\Rightarrow E_{R\lambda }=E_{T\lambda }+\Delta E_{M}.$$

Then

(40) $$1=\frac{E_{T\lambda }}{E_{R\lambda }}+\frac{\Delta E_{M}}{E_{R\lambda }}\Rightarrow \eta_{R}=1-{\bar{\eta }}_{R}$$

where *E_Tλ_*—theoretical (model) energy consumed to obtain the desired degree of fineness of plastics output material, *J*, ∆*E_M_*—change (increase) in energy losses for shredding only in machine conditions, *J*.

After some transformations, the change (increase) in energy losses for shredding in machine conditions can be expressed as a special coefficient [35]:

(41) $$E_{T\lambda }=\int_{t_{0}}^{T}p_{d}^{E}\left(t\right)dt\Rightarrow E_{T\lambda }=\alpha_{R}\cdot E_{R\lambda }$$

(42) $$\alpha_{R}=\eta_{R}=\frac{E_{T\lambda }}{E_{R\lambda }}$$

and

(43) $$\Delta E_{M}=\int_{t_{0}}^{T}p_{d}^{S}\left(t\right)dt\Rightarrow \Delta E_{M}=\beta_{R}\cdot E_{R\lambda }$$

(44) $$\beta_{R}={\bar{\eta }}_{R}=\frac{\Delta E_{M}}{E_{R\lambda }}$$

where *α_R_*—factor of energetic relations as a measure of model accomplishment in machine conditions (efficiency), *β_R_*—factor of energetic relations as a measure of machine conditions (inefficiency).

On the basis of previous assumptions and presented equations, it can be written [35]:

(45) $$\Delta E_{M}=E_{E}\cdot \eta_{n}-E_{T\lambda }.$$

This model includes energy dissipation; however, dissipation does not affect the breakage of polymeric material. On the basis of energy conservation law, the equation of shredding element kinetic energy before contact with the plastic material takes the following form [35]:

(46) $$E_{T\lambda }=E_{T}+E_{m}+E_{p}$$

or

(47) $$E_{T\lambda }=\frac{m_{1}\cdot v_{k}^{2}}{2}+\frac{m_{2}\cdot v_{k}^{2}}{2}+\left(1-k^{2}\right)\frac{m_{2}\cdot v_{1p}^{2}}{2}$$

where *E_T_*—kinetic energy of working element (phenomena) after the deformation of plastics grain, *J*, *E_m_*—kinetic energy of material particles after milling, *J*, *E_p_*—energy used for performing deformation work, *J, m*_1_—mass of working element, kg, *m*_2_—mass of plastics grain particles, kg, *v_k_*—ending velocity of working element and particles, m·s^−1^, *v*_1*p*_—beginning velocity of working element, m·s^−1^.

Assuming that the shredding force takes the following form [156]:

(48) $$E_{R\lambda }=P_{R}\cdot v_{R}\cdot t=\left(k_{j}\cdot v_{r}+\sigma_{\max}\cdot S_{r}+\varepsilon \cdot S_{r}^{\prime }\cdot v_{r}^{2}\right)\cdot v_{R}\cdot t$$

where *k_j_*—resistance coefficient of shredder running idle, kg·s^−1^, *v_r_*, *v_R_*—shredding velocity, m·s^−1^, (0.8–5.7 m·s^−1^), *σ*_max_—maximal stresses in the shredding area between cutting edges and disturbed polymeric materials particles, N·m^−2^, PP-waste: (16.45–21.87) MPa), *S_r_*—area of shredding field section, m^2^; $S_{r}^{\prime }$—proportion coefficient, N·s^2^·m^−4^;

The model of energy consumption during shredding was proposed [35]:

(49) $$E_{C}=E_{E}=\int_{t_{0}}^{T}p_{d}^{E}\left(t\right)dt-\int_{t_{0}}^{T}p_{d}^{S}\left(t\right)dt\Rightarrow E_{E}=\frac{\left(k_{j}\cdot v_{r}+\sigma_{\max}\cdot S_{r}+\varepsilon \cdot S_{r}^{\prime }\cdot v_{r}^{2}\right)\cdot v_{R}\cdot t}{\eta_{s}\cdot \eta_{p}}$$

where *t*—shredding time of the relative field section (*S_r_*), *s*. The energy model presented in Equation (36) contains the main elements of the energy-efficiency model of a multi-disc shredder.

The efficiency model of shredding technology including important factors of processing was described as follows [98]:

(50) $$e_{E}=f\left(P_{o},P_{e},P_{s},P_{od},E_{j},O_{nq}\right)$$

where *P_o_*—initial potential, *P_e_*—effectively used potential, *P_s_*—ineffectively lost potential, *P_od_*—potential recovered from technology or the surroundings, *E_j_*—unit energy consumption, *O_nq_*—low-quality product, waste, loss, defect operations, etc.

For polymer waste materials intended for energy use, the energy model including fuel-efficiency changes caused by shredding was proposed in [35]. It describes the overall efficiency of a multi-disc shredding process and takes the following form [35]:

(51) $$e_{R}=\frac{\int_{t_{0}}^{T}p_{O}^{o}\left(t\right)dt}{\int_{t_{0}}^{T}p_{O}^{U}\left(t\right)dt-\int_{t_{0}}^{T}p_{O}^{S}\left(t\right)dt}=\frac{\Delta \eta \cdot E_{brutto}}{E_{E}}\Rightarrow e_{r}=\frac{\left(\eta_{q-ś}-\eta_{m}\right)\cdot E_{brutto}\cdot \eta_{s}\cdot \eta_{p}}{\left(k_{j}\cdot v_{r}+\sigma_{\max}\cdot S_{r}+\varepsilon \cdot S_{r}^{\prime }\cdot v_{r}^{2}\right)\cdot v_{R}\cdot t}$$

which can be also presented in the form of an object-oriented relationship [35]:

(52) $$e_{r}=\frac{\eta_{q-s}\cdot E_{brutto}\cdot \eta_{s}\cdot \eta_{p}}{\left(k_{j}\cdot v_{r}+\tau_{q-ś}\cdot S_{q-ś}+\varepsilon \cdot S_{q-ś}^{\prime }\cdot v_{r}^{2}\right)\cdot v_{r}\cdot t}$$

where *ƞ_q−ś_*—factor of energy value, described on the ground of thermo-analysis for the shredding material, (for micro-polypropylene *ƞ_q−ś_* = 0.83), *ƞ_m_*—factor of plastics grain digestibility before shredding, (for polypropylene granulate grains *ƞ_m_* = 0.49), *E_brutto_*—energy contained in the PP-wastes being processed, MJ·kg^−1^ (PP-waste: (43–44) MJ·kg^−1^), *τ_q−ś_*—quasi-cutting stresses, N·m^−2^, *S_q−ś_*, $S_{q-ś}^{\prime }$—instantaneous and seconds cross-sectional PP-waste area of quasi-cutting, m^2^, *ƞ_q−s_*—material efficiency of the process of thermodynamic conversion of the quasi-cut product [35]:

(53) $$\eta_{q-s}=\frac{p_{em}^{E}}{p_{em}^{E}+p_{em}^{S}}\Rightarrow \eta_{q-ś}=\frac{{T^{\prime }}_{wy/q-ś}}{{T^{\prime }}_{we/q-ś}}$$

where ${T^{\prime }}_{wy/q-ś}$—output material mass after quasi-cutting, kg, ${T^{\prime }}_{we/q-ś}$—input material mass before quasi-cutting, kg, and *ƞ_m_*—material efficiency of the conversion process without quasi cutting of the material [35]:

(54) $$\eta_{m}=1-\eta_{q-s}\Rightarrow \eta_{m}=\frac{{T^{\prime }}_{wy}}{{T^{\prime }}_{we}}$$

where ${T^{\prime }}_{wy}$—output material mass subjected to quasi-cutting, kg, and ${T^{\prime }}_{we}$—input material mass without quasi-cutting, kg.

For calculation of the energy value for specific size classes of shredded polymer material, the factor of energy value *ƞ_q−ś_* was determined [35]:

(55) $$\eta_{q-s}=\int_{t_{0}}^{T}p_{d}^{o}\left(t\right)dt\Rightarrow \eta_{q-ś}=f_{<0.5}\eta_{q-ś,\;<0.5}+f_{0.5-1.5}\eta_{q-ś\;,0.5-1.5}+f_{>1.5}\eta_{q-ś,\;>1.5}$$

where *ƞ_q−ś_*_<0.5_, *ƞ_q−ś_*_0.5−1.5_, *ƞ_q−ś_*_>1.5_—factor of the energy value material described by its dimension, and *f*_<0.5_, *f*_0.5−1.5_, *f*_>1.5_—fraction share of the described dimension.

### 4.3. Control and Human Potentials (P^S^(t), P^L^(t))

The development of recycling potentials and effective action, including human development, depend on the knowledge and imagination of the creators. When implementing a monitoring concept adopted to increase the knowledge about the recycled and reused materials shredding process, the following must be chosen as part of the technical infrastructure selection procedure: information sources, types of measured quantities, interval values for measurement and measured data recording, and the implementation of simple and complex algorithms for aggregation of data. It is necessary to obtain reference values, which will be used for further analysis. Reports on test results must be pre-defined, for those interested in achieving the highest efficiency of the shredding process [71,149].

Considering the control potential (*L*) (Equation (56)), the motion, control, energy–environmental, and quality characteristics of multi-disc and multi-hole shredding process variables (power demand (*P_R_*)*,* fragmentation degree (*λ*), and production yield (*Q_m_*, *Q_C_*)) should depend on the following:

- The design features of the shredding unit (for instance, the common area of the edges of two holes (*S_c_,S_T_*)),
- Grain density and volume in the grinding chamber (*ρ_m_,V_g_*),
- Rotational, angular, and linear speed of a grinding component and time (respectively *n,ω,v*,Θ,*t_i_*) [148].

(56) $$L\left(P_{R},\lambda ,Q_{m},Q_{c}\right)=P\left(S_{c}^{\prime },S_{T},{\tilde{\rho }}_{n}^{m+1},V_{\bar{g}},n,\omega ,v,\Theta ,t_{i}\right)$$

The relationship to the control potential of the comminution system in recycling is as follows [190]:

(57) $$P^{S}\left(t\right)=\frac{S^{S}\pi^{S}\left(t\right)}{M^{S}\left(t\right)\varepsilon^{S}}$$

where

*S^S^*—possible control information stream,
*M^S^*(*t*)—a stream of used information,
*ε^S^*—theoretical possibilities and needs of information and decision systems,
*π^S^*—instantaneous actual stream of control information.

The control potential depends primarily on the exchange of information (including feedback) between the environment and the grinding system. The theoretical information capability *ε^S^* ideally is equal to 1 [190]:

(58) $$\varepsilon^{S}=\varepsilon_{a}^{S}\varepsilon_{e}^{S}\varepsilon_{d-d}^{S}\Rightarrow 1.$$

The conditions that must be met to achieve this state are as follows [70,190,191]:

- $\varepsilon_{a}^{S}=1$—information reaching and leaving the system ensure the implementation of autonomous, integral, and reliable operation,
- $\varepsilon_{e}^{S}=1$—the control system automatically neutralizes the negative effects of processing,
- $\varepsilon_{d-d}^{S}=1$—the control system is adapted to self-control and self-diagnosis within a predetermined tolerance field for effective system operation.

The control potential is related to the human potential, because the operators of machines, systems, and workstations can be part of the control system and be responsible for the flow of information transferred. The human potential for comminution processes in recycling was expressed by Flizikowski using the following relationship [190]:

(59) $$P^{L}\left(t\right)=\frac{L^{L}\pi^{L}\left(t\right)}{M^{L}\left(t\right)\varepsilon^{L}}$$

where

*L^L^*—number of people scheduled to carry out activities in the recycling (shredding) operations,
*M^L^*(*t*)—the number of people involved (taking part) in the recycling (shredding) operations,
*ε^L^*—theoretical human capabilities,
*π^L^*—the real value of human creativity and responsibility.

For one person provided for the implementation of tasks, human potential depends only on the theoretical human capabilities *ε^L^* and the real value of human creativity and responsibility *π^L^* [190]. As a rule, for the implementation of specific goals and activities, the real value of human creativity and responsibility *π^L^* should be in the range of 0.5–1.0 [190]. Values lower than 0.5 lead to the impossibility or incomplete implementation of the task [190]. The theoretical human capabilities *ε^L^* consists of the following [190]:

- Motivation to carry out entrusted tasks, when $\varepsilon_{m}^{L}=1$, one can talk about the ideal state of full motivation and full human commitment in the task,
- Knowledge about the entrusted task, when $\varepsilon_{w}^{L}=1$—full (complete) knowledge about the task,
- Access to self-improvement channels, when $\varepsilon_{k}^{L}=1$—open (full) access to self-improvement channels,
- Development of the market of goods and services adequate to meet human needs, when $\varepsilon_{r}^{L}=1$–, the market of goods and services is able to satisfy all needs of individuals.

The theoretical human capabilities reaches 1 when its components strive for one, which can be represented as [190]:

(60) $$\varepsilon^{L}=\varepsilon_{m}^{L}\varepsilon_{w}^{L}\varepsilon_{k}^{L}\varepsilon_{r}^{L}\Rightarrow 1.$$

## 5. Summary and Conclusions

This paper presents, organizes, and systematizes the issues related to the grinding of disturbed: *d*(*m*), *d*(*f*), *d*(*T*), *d*(*mp*) etc. polymer materials in recycling. Based on the analysis of the literature and statistics on polymer materials, it has been shown that up to 100% of polymer wastes are shredded. Shredding is a key process in the recycling and EoL management of elements and multi-plastic parts of machines. Thanks to shredding, it is possible to separate materials.

Cutting is the dominant method of comminution with respect to polymeric materials. The quasi-cutting phenomenon is used in popular multi-disc and drum shredders. This phenomenon is mainly determined by material properties (shear strength, cutting resistance), machine properties (geometric features of the working unit, strength properties of the working unit, motion resistance), and process parameters (cutting speed, medium share, dosed material flow).

The most important indicators for the shredding process evaluation were presented in this study. They can be used to improve and develop shredding processes in recycling, especially in the use of human, technological, energy–material, and control potentials. The key to the comminution process is the introduction of active, automatic, intelligent control systems aimed at minimizing the environmental loads (for instance, minimizing the consumption of energy and raw materials, the amount of waste, the emission of dust and harmful oxides) and maximizing the effects (i.e., efficiency, also targeted at obtaining a product with desired size). The literature analysis shows that the main challenge for the comminution line is to reduce the process energy consumption.

Presented bases, models, and factors affecting the shredding of polymer materials and material motion, for idle run and a loaded shredding chamber, could be used for the development and improvement of shredding machines. The need for reaching a compromise between the two basic functions—movement and shredding in the multi-hole, multi-disc grinding unit—should be pointed out on the basis of a literature review under material motion in the shredders. It is a useful and desired course, resulting ultimately in obtaining a high-quality product with a defined form, structure, and repeatable dimensions.

Models and corresponding mathematical dependencies facilitate the efficient designing and planning of multi-hole shredding systems utilization. Procedures for active, environmental, and compensatory monitoring of shredding parameters should be formulated for the purpose of the eco-design of special systems machines, devices, and lines for shredding in the polymeric materials recycling. The described dependencies that enable the identification of product quality in technology and process control based on the product quality indicators, the actual state of effectiveness of the mechanical processing, and its harmlessness can support the decision makers and designers of the recycling technology. The provided relations between shredding process parameters, energy consumption, and product quality can be used in the industry sector for controlling the shredding processes. Presented procedures can be used to support innovation and creative activities, i.e., environmentally friendly actions, which lead to achieving specific positive environmental results in the recycling and the mechanical processing of recycled and reused materials.

The literature survey indicates the need to explore the environmental aspects of the shredding process in recycling and connect the shredding process variables with environmental consequences. This will help design and control the processes to get the possibly lowest environmental burdens.

## Figures and Tables

**Table 1:** Estimated scale of shredding EoL polymer materials in EU countries in 2018.

End-Of-Life Option	Total Amount of Waste ^1^, mln t	Percentage of Waste Shredded ^2^, %	Mass of Waste Shredded, mln t
Landfill	7.2	2	0.144
Energy Recovery	12.4	20	2.480
Recycling	9.5	50 (100 ^3^)	4.750 (9.500)
Sum	29.1	-	7.374 (12.124)

^1^ data based on Eurostat [78], ^2^ data based on [48], ^3^ data based on [81].

**Table 2:** Shredding types (methods) according to [36,99,100,101].

Type	Load Model	Stresses
Crushing	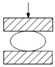	Compressive stresses
Shear	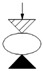	Shear stresses
Abrasion	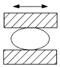	Surface pressures
Impact	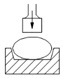	Surface pressures
Breaking	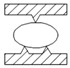	Bending stresses

**Table 3:** Types of shredding depending on the grinding product size class according to [46].

Type	Grain Size Classes
coarse crushing	50–500 mm
fine crushing	5–50 mm
very fine crushing	0.5–5 mm
coarse grinding	0.1–1 mm
fine grinding	10–150 µm
very fine grinding	1–20 µm
colloidal grinding	0.1–2 µm

**Table 4:** The investigations on quasi-static strength terms presented in [133].

Stage of the Problem	Results & Solution
Strength-static investigations of PVC post-use pipe carried out by INSTRON 8501—Value of energy needed to disintegration (lines blade angle *β* = 60°, *β* = 75°, *β* = 90°, *β* = 105°, *β* = 120°—models of deformation and loads of PVC pipe)	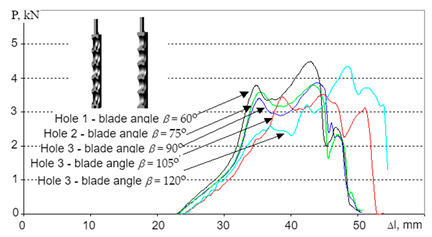 *P* = *f* (Δ*l*), *p*-value of force, Δ*l*—value of displacement
Unevenness of quasi-shear forces *P* and plate displacements, for different sample (disturbed) forms and settings in the instrument. Blade angle *β* = 90°, plastic: Low-density polyethylene (LDPE) pipe, outer diameter *D_z_* = 40 mm, wall thickness, *g* = 4.3 mm, sample length *l_p_* = 50 mm, relative speed of shredding *v_r_* = 30 mm·s^−1^	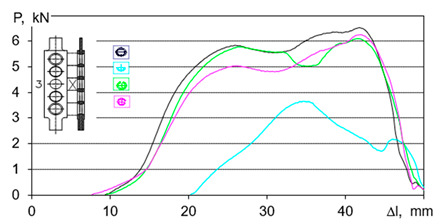 *P* = *f* (Δ*l*), *p*-value of force, Δ*l*—value of displacement

**Table 5:** Selected research areas on the multi-disc grinding process in the last 25 years.

Lp.	Research Area	Ref.
1	Determination of the dependencies and effects of the shredder design features and the charge physical and mechanical features on the grinding parameters, i.e., energy consumption and quality	[164]
2	Study of the multi-disc grinders uneven operation	[162,163]
3	Study of the grinding process efficiency in a supersonic disc grinder	[165]
4	Study of the influence of inter-disc gap size on the grinding product quality, energy consumption, and efficiency	[166]
5	Analysis of energy losses in the form of heat during grinding and the possibility of its recovery	[167]
6	Dynamic analysis of forces acting on the shredded material and the grinding disc	[152]
7	Investigation of the influence of the grinding process parameters on the product physical properties and microstructure	[168]

**Table 6:** Grinding indicators classification according to Macko [173].

GRINDING INDICATORS		
Technological	Technical	Economic
Size reduction ratio	Specific energy consumption	Operating costs
Specific surface area	Possibility of cooperation with other devices	Investment costs
The degree of surface growth	Effectiveness	Costs of accompanying processes
Total efficiency		
Grain shape		

**Table 7:** Grinding indicators classification according to Flizikowski [45,174].

GRINDING INDICATORS		
Quality	Efficiency	Harmlessness
Product (size reduction ratio)	Energy	Pollution emission indicators
Process (Efficiency)	Economic	Noise emission indicators
Machine	Ecological	Waste emission indicators

## Data Availability

No new data were created or analyzed in this study. Data sharing is not applicable to this article.
